# MHD mixed convection of hybrid nanofluid in a wavy porous cavity employing local thermal non-equilibrium condition

**DOI:** 10.1038/s41598-021-95857-z

**Published:** 2021-08-25

**Authors:** Zehba Raizah, Abdelraheem M. Aly, Noura Alsedais, Mohamed Ahmed Mansour

**Affiliations:** 1 Department of Mathematics, College of Science, King Khalid University, Abha, 62529 Saudi Arabia; 2 Department of Mathematics, Faculty of Science, South Valley University, Qena, 83523 Egypt; 3 Department of Mathematical Sciences, College of Science, Princess Nourah Bint Abdulrahman University, Riyadh, Saudi Arabia; 4 Mathematics Department, Faculty of Science, Assiut University, Assiut, Egypt

**Keywords:** Applied mathematics, Computational science

## Abstract

The current study treats the magnetic field impacts on the mixed convection flow within an undulating cavity filled by hybrid nanofluids and porous media. The local thermal non-equilibrium condition below the implications of heat generation and thermal radiation is conducted. The corrugated vertical walls of an involved cavity have ${T}_{c}$ and the plane walls are adiabatic. The heated part is put in the bottom wall and the left-top walls have lid velocities. The controlling dimensionless equations are numerically solved by the finite volume method through the SIMPLE technique. The varied parameters are scaled as a partial heat length (*B*: 0.2 to 0.8), heat generation/absorption coefficient (*Q*: − 2 to 2), thermal radiation parameter (*R*_*d*_: 0–5), Hartmann number (*Ha*: 0–50), the porosity parameter (*ε*: 0.4–0.9), inter-phase heat transfer coefficient (H^*^: 0–5000), the volume fraction of a hybrid nanofluid (*ϕ*: 0–0.1), modified conductivity ratio (*k*_*r*_: 0.01–100), Darcy parameter $\left(Da: 1{0}^{-1}\,\mathrm{ to }\,1{0}^{-5}\right)$, and the position of a heat source (*D*: 0.3–0.7). The major findings reveal that the length and position of the heater are effective in improving the nanofluid movements and heat transfer within a wavy cavity. The isotherms of a solid part are significantly altered by the variations on $Q$, ${R}_{d}$, ${H}^{*}$ and ${k}_{r}$. Increasing the heat generation/absorption coefficient and thermal radiation parameter is improving the isotherms of a solid phase. Expanding in the porous parameter $\varepsilon$ enhances the heat transfer of the fluid/solid phases.

## Introduction

In computational fluid dynamics (CFD) mechanism, it has been applied extensively to examine the flow specifics and are characterized methods for scheming and optimizing systems at a wide range of imitative engineering applications^1–3^. The simulation of heat and mass transfer in cavities is one of the important and interesting research fields in CFD. One of the applications of simulating the convection flow of heat and mass transfer inside moving wavy wall cavities, is the mixing in human digestive organs, such as the stomach and intestine, whose walls keep moving during the digestion period^4–6^. This method is completely favorable to analyze the mixing operation in wavy wall cavities to discover different influencing operators.

Hybrid nanofluid is a blend of two distinct forms of nanoparticles distributed in a host fluid. Suresh et al.^7^ investigated experimentally the properties of hybrid nanofluids and discovered that an agreeable mixture of selected nanoparticles may enhance each other's positive features. Tayebi and Chamkha^8^ investigated how a hybrid nanofluid could boost natural convection with an annulus. When compared to nanofluid, it was discovered that using a hybrid nanofluid is more efficient. Chamkha et al.^9^ studied the influences of a magnetic field on hybrid nanomaterial enclosed by two surfaces. Further numerical attempts in natural/mixed convection of a hybrid nanofluid can be consulted by the Refs.^10–16^.

Natural/mixed convection with a magnetic field in open/closed cavities is of great importance in industrial applications for instance polymer and metallurgy, geothermal energy extraction, and fusion reactors. Then, this topic has been investigated in several studies^17–30^. Sheremet et al.^31^ examined the MHD free convection of a nanofluid in an open porous high cavity with a corner heater. Hamid and Shahriari^32^ used a wavy-walled open cavity filled with a hybrid nanofluid. In an important study, Li et al.^33^ reported that the larger amplitude and higher frequency of the moving wall can efficiently mix the viscous fluid. Also, Zou et al.^34^ summarized that the flow direction should be considered to promote convective mixing.

The heat equation has two different models for the two phases of a porous matrix. The first model is a local thermal equilibrium (LTE), which undertakes all solid and fluid temperatures are similar. When the temperatures of the solid and fluid are differing, the LTE model does not approach. The second model is a local thermal non-equilibrium (LTNE). There are still limited studies using the LTNE approach for porous media. Baytas and Pop^35^ analyzed numerically the free convection of nanofluid within a cavity by adopting LTNE, where Bhadauria and Agarwal^36^ analyzed the consequence of LTNE on the thermal instability in porous media. Under the condition of LTNE, Alsabery et al.^37^ explored the thermal natural convective of a nanofluid in a wavy domain saturated by non-Darcian porous media. Mansour et al.^38^ researched the magnetic field and LTNE model impacts on the Marangoni convective flows of a micropolar nanofluid inside an open enclosure.

From the previous studies and to our expertise, no related studies on the mixed convection in an inclined wavy cavity filled by porous media and hybrid nanofluids under the LTNE condition. Accordingly, the research target is to evaluate the mixed convection flow of water, copper, and titanium dioxide (TiO_2_) in the interior of an inclined wavy cavity drenched by a porous medium. The obtained results indicated that the length/position of the partial heater is adapting the characteristics of the nanofluid flow and heat transfer inside a wavy cavity. The involved parameters in the thermal solid-phase equation such as inter-phase heat transfer coefficient ${H}^{*}$, heat generation/absorption coefficient, thermal radiation parameter, and modified conductivity ratio ${k}_{r}$ have slight effects on the isotherms of a fluid phase and streamlines, whilst the isotherms of a solid part are significantly affected by the variations on these parameters. The lower Darcy parameter provides a reduction in the flow speed and enhances the isotherms within a wavy cavity. Increasing Hartmann number from 0 to 50 reduces the streamlines’ maximum by $26.32\%$. In sum, the main goal of this study is to investigate the potential factors that could enhance the heat transfer inside the moving wavy cavities for taking the advantage of the numerical results in simulating the fluid flow inside mixing vessels and reactors or digesters inside living organisms. Further, the motion of the wavy wall can be practiced inside an in-plane wall motion^39^.

## Mathematical formulation

**Figure 1:**
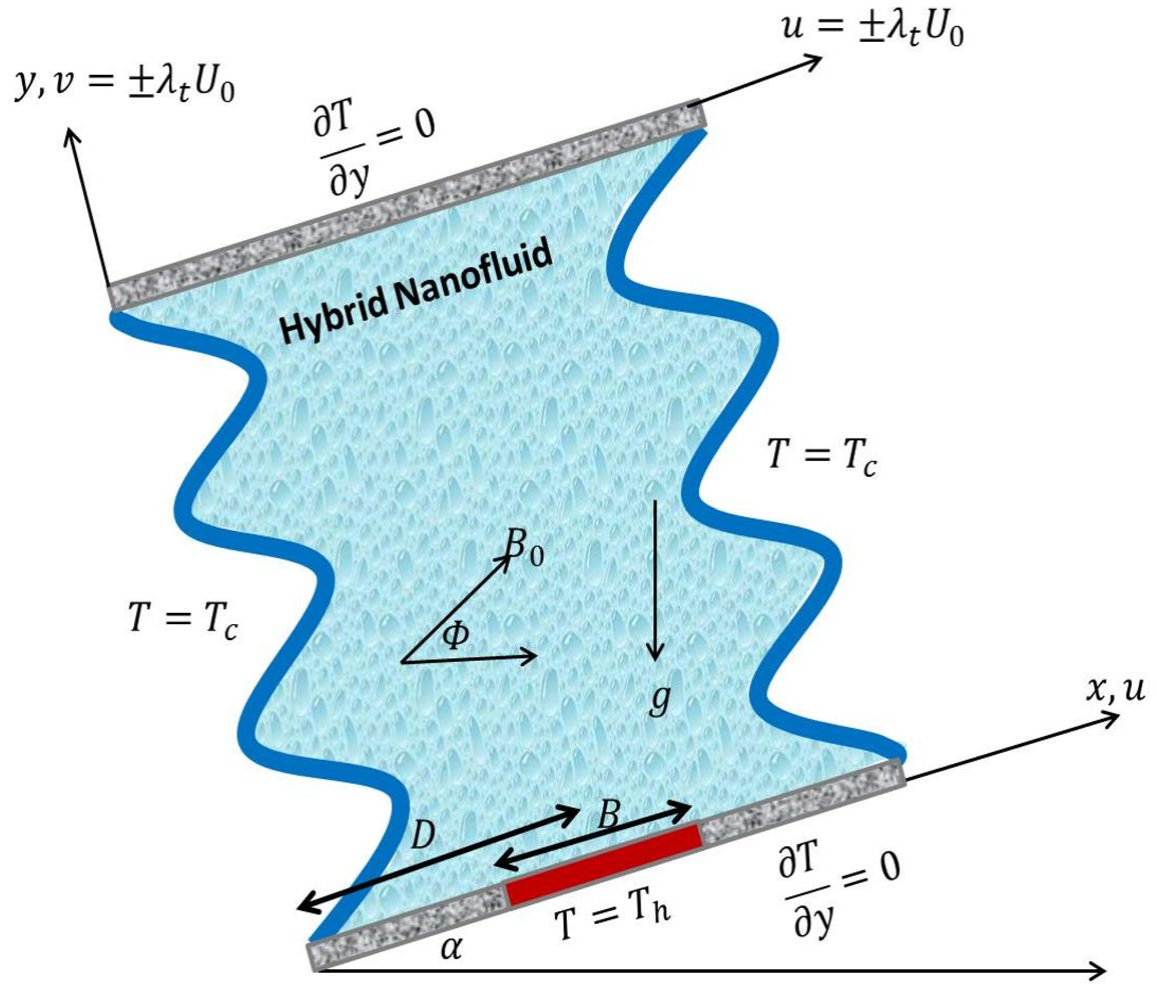
Initial geometry of an inclined wavy cavity.

Figure 1 shows the preliminary geometry of an inclined undulating cavity. The involved wavy cavity is filled by porous media and hybrid nanofluids. A partial heat source is laid in the bottom wall with a variable-length $B$ and the other horizontal walls are adiabatic. The vertical sidewalls are cold (${T}_{c}$), the left wavy and top walls have lid velocities. The magnetic field has an inclination angle $\Phi$. The undulating cavity is inclined by an inclination angle $\alpha$. The hybrid nanofluid convection is not in a local thermodynamic equilibrium condition. The normal direction and constant value are considered for the gravity acceleration. Dirichlet type applied on all boundaries (no-slip condition). Considering the earlier specified hypotheses, the continuity, momentum, and energy equations concerning the hybrid nanofluid, incompressible, laminar, single-phase, and steady-state flow are formulated as follows^40,41^:

1 $$\frac{\partial u}{\partial x}=-\frac{\partial v}{\partial y}$$

2 $$\frac{u}{{\varepsilon }^{2}}\frac{\partial u}{\partial x}+\frac{v}{{\varepsilon }^{2}}\frac{\partial u}{\partial y}=-\frac{1}{{\rho }_{hnf}}\frac{\partial p}{\partial x}+\frac{1}{\varepsilon }\cdot {\nu }_{hnf}\cdot {\nabla }^{2}u-\frac{{\nu }_{hnf}}{K}\cdot u+\frac{(\rho \beta {)}_{hnf}}{{\rho }_{hnf}}g({T}_{f}-{T}_{c})\mathit{sin}\alpha +\frac{{\sigma }_{hnf}{B}_{0}^{2}}{{\rho }_{hnf}}(v\mathit{sin}\Phi \mathit{cos}\Phi -u{\mathit{sin}}^{2}\Phi ),$$

3 $$\frac{u}{{\varepsilon }^{2}}\frac{\partial v}{\partial x}+\frac{v}{{\varepsilon }^{2}}\frac{\partial v}{\partial y}=-\frac{1}{{\rho }_{hnf}}\frac{\partial p}{\partial y}+\frac{1}{\varepsilon }\cdot {\nu }_{hnf}\cdot {\nabla }^{2}v-\frac{{\nu }_{hnf}}{K}v+\frac{(\rho \beta {)}_{hnf}}{{\rho }_{hnf}}g({T}_{f}-{T}_{c})\mathit{cos}\alpha +\frac{{\sigma }_{hnf}{B}_{0}^{2}}{{\rho }_{hnf}}(u\mathit{sin}\Phi \mathit{cos}\Phi -v{\mathit{cos}}^{2}\Phi ),$$

4 $$\frac{1}{\varepsilon }\left(u\frac{\partial {T}_{f}}{\partial x}+v\frac{\partial {T}_{f}}{\partial y}\right)={\alpha }_{eff\cdot hnf}\cdot {\nabla }^{2}{T}_{f}+\frac{{h}_{nfs}\left({T}_{s}-{T}_{f}\right)}{\varepsilon (\rho {c}_{p}{)}_{hnf}}+\frac{{Q}_{0}\left({T}_{f}-{T}_{c}\right)}{\varepsilon (\rho {c}_{p}{)}_{hnf}},$$

5 $$0=(1-\varepsilon )\left({k}_{s}+\frac{16{\sigma }^{*}{T}_{c}^{3}}{3{k}^{*}}\right){\nabla }^{2}{T}_{s}+{h}_{nfs}({T}_{f}-{T}_{s})+(1-\varepsilon ){Q}_{0}({T}_{f}-{T}_{c}),$$

where $u$ and $v$ are the velocity components, $T$ is a temperature, ${\rho }_{hnf}$ is the density, ${\nu }_{hnf}$ is kinematic viscosity. $g$ is a gravity, $p$ is a pressure, ${\mu }_{hnf}$ is a dynamic viscosity. ${Q}_{0}$ is the heat generation $({Q}_{0}>0)$ or absorption $({Q}_{0}<0)$ coefficient.

Introducing the dimensionless set as:

6 $$X=\frac{x}{H},Y=\frac{y}{H},U=\frac{u}{{U}_{0}},V=\frac{v}{{U}_{0}},P=\frac{p}{{\rho }_{nf}{U}_{0}^{2}},{\theta }_{f}=\frac{({T}_{f}-{T}_{c})}{\Delta T},{\theta }_{s}=\frac{({T}_{s}-{T}_{c})}{\Delta T},$$

$$Ri=\frac{Gr}{{Re}^{2}}$$, $$\Delta T=({T}_{h}-{T}_{c}),D=d/H,B=b/H$$

The subsequent dimensionless equations when substituting Eq. (6) into Eqs. (1)–(5) are:

7 $$\frac{\partial U}{\partial X}+\frac{\partial V}{\partial Y}=0,$$

8 $$\begin{aligned} \frac{1}{{\varepsilon ^{2} }}\left( {U\frac{{\partial U}}{{\partial X}} + V\frac{{\partial U}}{{\partial Y}}} \right) = & - \frac{{\partial P}}{{\partial X}} + \frac{1}{{\varepsilon \text{Re} }}\left( {\frac{{\rho _{f} }}{{\rho _{{hnf}} }}} \right)\left( {\frac{{\mu _{{hnf}} }}{{\mu _{f} }}} \right)\nabla ^{2} U - \frac{1}{{Da\text{Re} }}\left( {\frac{{\rho _{f} }}{{\rho _{{hnf}} }}} \right)\left( {\frac{{\mu _{{hnf}} }}{{\mu _{f} }}} \right)U \\ & \; + Ri\frac{{(\rho \beta )_{{hnf}} }}{{\rho _{{hnf}} \beta _{f} }}\theta _{f} sin\,\alpha \,\, + \left( {\frac{{\rho _{f} }}{{\rho _{{hnf}} }}} \right)\left( {\frac{{\sigma _{{hnf}} }}{{\sigma _{f} }}} \right)\frac{{Ha^{2} }}{{\text{Re} }}(V\sin \Phi \cos \Phi - U\sin ^{2} \Phi ) \\ \end{aligned}$$

9 $$\begin{aligned} \frac{1}{{\varepsilon^{2} }}\left( {U\frac{\partial V}{{\partial X}} + V\frac{\partial V}{{\partial Y}}} \right) = & - \frac{\partial P}{{\partial Y}} + \frac{1}{{\varepsilon {\text{Re}} }}\left( {\frac{{\rho_{f} }}{{\rho_{hnf} }}} \right)\left( {\frac{{\mu_{hnf} }}{{\mu_{f} }}} \right)\nabla^{2} V - \frac{1}{{Da{\text{Re}} }}\left( {\pi \frac{{\rho_{f} }}{{\rho_{hnf} }}} \right)\left( {\pi \frac{{\mu_{hnf} }}{{\mu_{f} }}} \right)V \\ & + Ri\frac{{(\rho \beta )_{hnf} }}{{\rho_{hnf} \beta_{f} }}\theta_{f} cos\,\alpha \,\, + \left( {\frac{{\rho_{f} }}{{\rho_{hnf} }}} \right)\left( {\frac{{\sigma_{hnf} }}{{\sigma_{f} }}} \right)\frac{{Ha^{2} }}{{\text{Re}}}(U\sin \Phi \cos\, \Phi - V\cos^{2} \Phi ), \\ \end{aligned}$$

10 $$\begin{aligned} \frac{1}{\varepsilon }\left( {U\frac{{\partial \theta_{f} }}{\partial X} + V\frac{{\partial \theta_{f} }}{\partial Y}} \right) =& \left( {\frac{1}{Re\Pr }} \right)\frac{{\alpha_{eff.nf} }}{{\alpha_{f} }}\nabla^{2} \theta_{f} \,\, + \,\,\frac{1}{{\varepsilon {\text{Re}} \Pr }}\frac{{(\rho \,c_{p} )_{f} }}{{(\rho \,c_{p} )_{nf} }}H^{*} (\theta_{s} - \theta_{f} ) \\ & + \frac{1}{{\varepsilon {\text{Re}} \Pr }}\frac{{(\rho \,c_{p} )_{f} }}{{(\rho \,c_{p} )_{nf} }}Q\theta_{f} , \\ \end{aligned}$$

11 $$0=(1+{R}_{d}){\nabla }^{2}{\theta }_{s}+{K}_{r}{H}^{*}({\theta }_{f}-{\theta }_{s})+{k}_{fs}Q{\theta }_{f},$$

where $$\mathit{Pr}=\frac{{\nu }_{f}}{{\alpha }_{f}},\mathit{Re}=\frac{{U}_{0}H}{{\nu }_{f}},Gr=\frac{g{\beta }_{f}{H}^{3}\Delta T}{{{\nu }^{2}}_{f}},\mathit{Ha}={B}_{0}H\sqrt{{\sigma }_{f}/{\mu }_{f}},$$$$Da=\frac{K}{{H}^{2}},{R}_{d}=\frac{16{\sigma }^{*}{T}_{c}^{3}}{3{k}^{*}{k}_{s}},{H}^{*}={h}_{nfs}\frac{{H}^{2}}{{k}_{f}},Q={Q}_{0}\frac{{H}^{2}}{{k}_{f}},{k}_{\mathit{fs}}=\frac{{k}_{f}}{{k}_{s}},{K}_{r}=\frac{{k}_{f}}{(1-\varepsilon ){k}_{s}},$$

Corresponding boundary conditions:

On a left wall

12 $$U=0,V=\pm {\lambda }_{t},\theta_{f}=\theta_{s}=0:X=A\left[1-\mathit{cos}\left(2\pi \lambda Y\right)\right],0\le Y\le 1$$

On a right wall

13 $$U=0,V=0, \theta_{f}=\theta_{s}=0:X=1-A\left[1-\mathit{cos}\left(2\pi \lambda Y\right)\right],0\le Y\le 1$$

On a top wall

14 $$U=\pm {\lambda }_{t},V=0,\frac{\partial \theta_{f} }{\partial Y}=\frac{\partial \theta_{s} }{\partial Y}=0: 0\le X\le 1,Y=1$$

On a bottom wall

15 $$U=V=0, \theta_{f}=\theta_{s} =1: Y=0, D-0.5B \le X \le D+0.5B, U=V=0, \frac{\partial \theta_{f} }{\partial Y} = \frac{\partial \theta_{s}}{\partial Y}=0: Y=0, X \le D-0.5B \, or \, X \ge D+0.5B $$

The local Nusselt number is defined as:

16 $$Nu_{{fs}} = - \frac{{k_{{eff.hnf}} }}{{k_{{eff.f}} }}\left( {\frac{{\partial \theta _{f} }}{{\partial Y}}} \right)_{{Y = 0}}$$

17 $$Nu_{{ss}} = - (1 + R_{d} )\left( {\frac{{\partial \theta _{s} }}{{\partial Y}}} \right)_{{Y = 0}}$$

And average Nusselt number is:

18 $$N{u}_{mf}=\frac{1}{B}{\int }_{D-0.5B}^{D+0.5B}N{u}_{fs}dX$$

19 $$N{u}_{ms}=\frac{1}{B}{\int }_{D-0.5B}^{D+0.5B}N{u}_{ss}dX$$

## Hybrid nanofluid

The effective thermal diffusion and conductivity are:

20 $${\alpha }_{eff,hnf}=\frac{{k}_{eff,hnf}}{{\left(\rho {c}_{p}\right)}_{hnf}}$$

21 $${\alpha }_{eff,f}=\frac{{k}_{eff,f}}{{\left(\rho {c}_{p}\right)}_{f}}$$

22 $${k}_{eff,hnf}=\varepsilon {k}_{hnf}+\left(1-\varepsilon \right){k}_{s}$$

23 $${k}_{eff,f}=\varepsilon {k}_{f}+\left(1-\varepsilon \right){k}_{s}$$

Thermal diffusivity is:

24 $$\frac{{\alpha }_{hnf}}{{\alpha }_{f}}=\frac{\frac{{k}_{hnf}}{{k}_{f}}}{\frac{{\left(\rho {c}_{p}\right)}_{hnf}}{{\left(\rho {c}_{p}\right)}_{f}}}$$

Effective density is:

25 $$\frac{{\rho }_{hnf}}{{\rho }_{f}}=\left(1-{\phi }_{Cu}\right)\left(1-{\phi }_{Ti{O}_{2}}+{\phi }_{Ti{O}_{2}}\frac{{\rho }_{Ti{O}_{2}}}{{\rho }_{f}}\right)+{\phi }_{Cu}\frac{{\rho }_{Cu}}{{\rho }_{f}}$$

The heat capacitance is:

26 $$\frac{{\left(\rho {C}_{p}\right)}_{hnf}}{{\left(\rho {C}_{p}\right)}_{f}}=\left(1-{\phi }_{Cu}\right)\left(1-{\phi }_{Ti{O}_{2}}+{\phi }_{Ti{O}_{2}}\frac{{\left(\rho {C}_{p}\right)}_{Ti{O}_{2}}}{{\left(\rho {C}_{p}\right)}_{f}}\right)+{\phi }_{Cu}\frac{{\left(\rho {C}_{p}\right)}_{Cu}}{{\left(\rho {C}_{p}\right)}_{f}}$$

The thermal expansion is:

27 $$\frac{{\beta }_{hnf}}{{\beta }_{f}}=\left(1-{\phi }_{Cu}\right)\left(1-{\phi }_{Ti{O}_{2}}+{\phi }_{Ti{O}_{2}}\frac{{\beta }_{Ti{O}_{2}}}{{\beta }_{f}}\right)+{\phi }_{Cu}\frac{{\beta }_{Cu}}{{\beta }_{f}}$$

The thermal conductivity is:

28 $$\frac{{k}_{hnf}}{{k}_{bf}}=\frac{{k}_{Cu}+2{k}_{bf}-2{\phi }_{Cu}\left({k}_{bf}-{k}_{Cu}\right)}{{k}_{Cu}+2{k}_{bf}+{\phi }_{Cu}\left({k}_{bf}-{k}_{Cu}\right)}$$

where $$\frac{{k}_{bf}}{{k}_{f}}=\frac{{k}_{Ti{O}_{2}}+2{k}_{f}-2{\phi }_{1}\left({k}_{f}+{k}_{Ti{O}_{2}}\right)}{{k}_{Ti{O}_{2}}+2{k}_{f}+{\phi }_{1}\left({k}_{f}-{k}_{Ti{O}_{2}}\right)}$$

The effective dynamic viscosity is:

29 $$\frac{{\sigma }_{hnf}}{{\sigma }_{bf}}=\frac{{\sigma }_{Cu}+2{\sigma }_{bf}-2{\phi }_{Cu}\left({\sigma }_{bf}-{\sigma }_{Cu}\right)}{{\sigma }_{Cu}+2{\sigma }_{bf}+{\phi }_{Cu}\left({\sigma }_{bf}-{\sigma }_{Cu}\right)}$$

where $$\frac{{\sigma }_{bf}}{{\sigma }_{f}}=\frac{{\sigma }_{Ti{O}_{2}+}2{\sigma }_{f}-2{\phi }_{Ti{O}_{2}}\left({\sigma }_{f}-{\sigma }_{Ti{O}_{2}}\right)}{{\sigma }_{Ti{O}_{2}+}2{\sigma }_{f}+{\phi }_{Ti{O}_{2}}\left({\sigma }_{f}-{\sigma }_{Ti{O}_{2}}\right)}$$


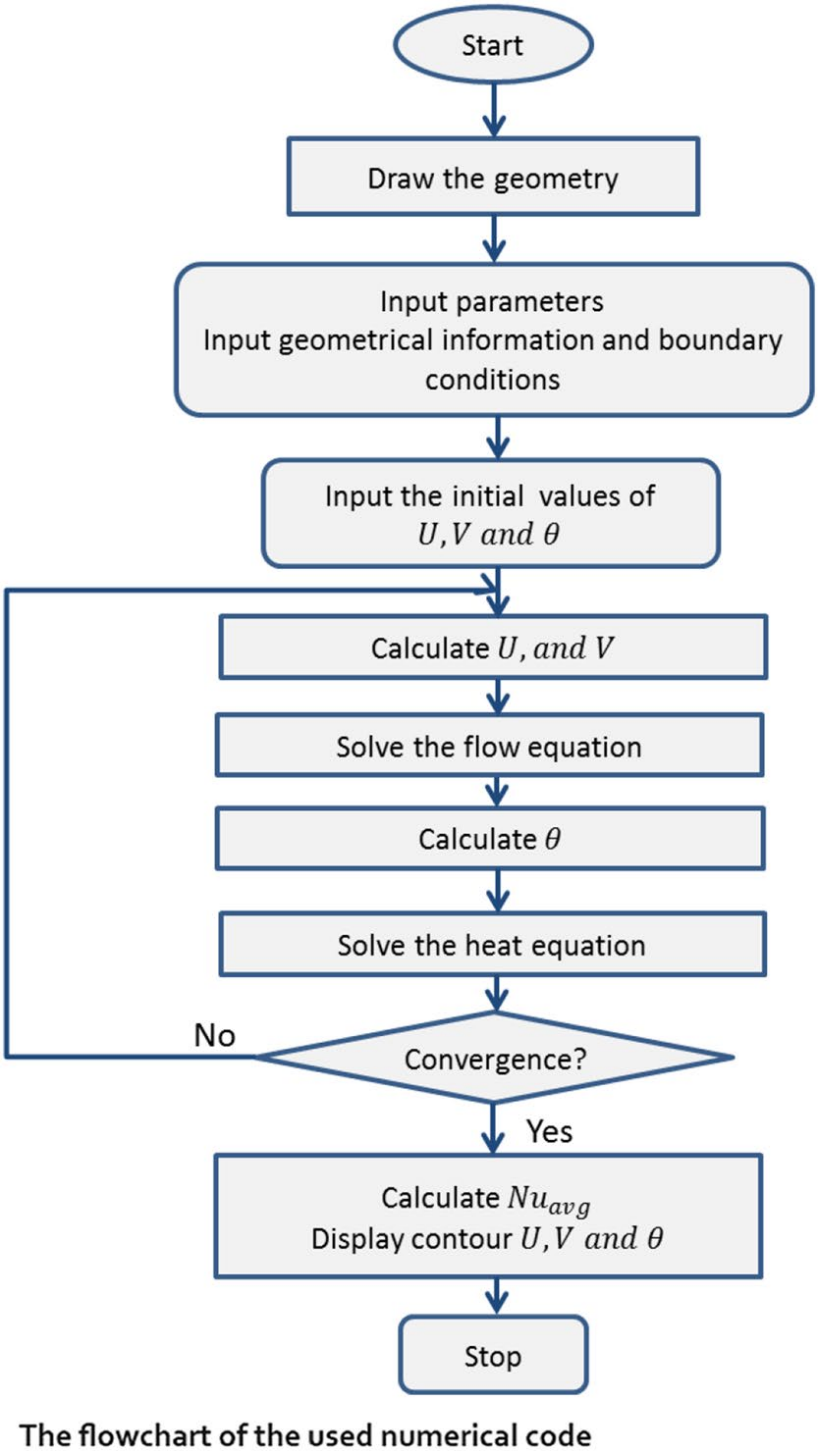


## Numerical method

In this study, the finite volume method (FVM) based on the SIMPLE algorithm^44^ is applied to solve the governing equations. The system of the governing Eqs. (7)–(11) corresponding to the boundary conditions (12)–(15) is written in the following form (Table 1):

30 $$\underset{\Omega }{\overset{ }{\int }} \left(\widehat{u}\Phi -{\Gamma }_{\Phi } grad\Phi \right)\cdot \widehat{n}d\mathrm{S}=\underset{\Omega }{\overset{ }{\int }}{S}_{\Phi }dV,$$

where $\phi$ refers to $U,V, {\theta }_{f}\, \text{ and }\, {\theta }_{s}$ and $\Omega$ refers to the control volume. The first upwind scheme is used for the advection term and the central differences approach is applied for the diffusive fluxes; then the following algebraic system is obtained:

31 $$\sum_{faces }{\widehat{u}}_{f}{\Phi }_{f}{A}_{f}-\sum_{faces}{{\Gamma }_{\Phi }}_{f}{\left(\nabla\Phi \right) }_{\perp ,f}{A}_{f}-{S}_{\Phi }V=0,$$

where A is the area of the cell and f refers to the faces. Here the convergence criteria are ${10}^{-6}$.

**Table 1:** Physical attributes of water, copper, and titanium dioxide^42,43^.

	Water	Copper	TiO_2_
$\rho \left(\frac{kg}{{m}^{3}}\right)$	997.1	8933	4250
${C}_{p}\left(\frac{J}{kgK}\right)$	4179	385	686.2
$k\left(\frac{W}{mK}\right)$	0.613	401	8.9538
${\beta }_{T}\times 1{0}^{-5}\left(\frac{1}{K}\right)$	21	1.67	0.9
$\sigma \left(S/m\right)$	0.05	$5.96\times 1{0}^{-7}$	$2.38\times 1{0}^{6}$

Many grid tests are carried out and presented in Table 2 and it is found that the grid size of $141\times 141$ is appropriate for all the computations. Moreover, no special treatment on the grids of the curved walls is considered. Almost uniform grids are adopted for all the geometry.

**Table 2:** Grid independence study using average Nusselt number (Nu) at $a=10, {R}_{d}=0.5, Gr=1{0}^{3},\mathrm{ and }\, Ri=1$.

Grid size	$41\times 41$	$61\times 61$	$81\times 81$	$101\times 101$	$121\times 121$	$141\times 141$	$161\times 161$
Nu_mf_	23.81320	17.14709	17.56104	17.64019	17.66233	17.66508	17.65852
Nu_ms_	5.314915	5.32550	5.372007	5.395264	5.410059	5.420341	5.428008

Also, Table 3 shows the comparison of the average Nusselt Number for the different values Hartmann number $Ha$. From this comparison, it is seen that the present results from the finite volume method agree well with the results from Biswas and Manna^45^.

**Table 3:** Comparison of the average Nusselt Number for the different values Hartmann number $Ha$.

$Ha$	Assisting flow (downward lid motion)		%Errors	opposing flow (upward lid motion)		%Errors
	Present results	Biswas and Manna^45^		Present results	Biswas and Manna^45^	
10	10.361	10.676	2.951	10.653	10.307	− 3.357
50	10.314	10.302	− 0.116	10.337	10.276	− 0.594

An in-house code of the FVM with SIMPLE scheme is written in FORTRAN-90. The calculations are performed by SHAHEEN-II Cluster managed by King Abdullah University of Science and Technology (KAUST), Jeddah, Saudi Arabia.

## Results and discussion

The research treats the numerical flow of hybrid nanofluids motivated by mixed convection in a wavy inclined cavity with LTNE condition and saturated by porous media. The scales of the varied parameters are partial heat length $B=0.2-0.8$, heat generation/absorption coefficient $Q=-2-2$, thermal radiation parameter ${R}_{d}=0-5$, Hartmann number $Ha=0-50$, the porosity parameter $\varepsilon =0.4-0.9$, inter-phase heat transfer coefficient ${H}^{*}=0-5000$, the volume fraction of a hybrid nanofluid $\upphi =0-0.1$, modified conductivity ratio ${k}_{r}=0.01-100$, Darcy parameter $Da=1{0}^{-1}-1{0}^{-5}$, and the position of a heat source $D=0.3-0.7$. The fixed parameters are the Grashof parameter $Gr=1{0}^{3},$ an inclination angle of a cavity $\alpha =\pi /4,$ amplitude parameter $A=0.1$, angle of a magnetic field $\Phi =\pi /3$, Richardson parameter $Ri=1$, lid-velocity ${\lambda }_{t}={\lambda }_{l}=1$, and a phase deviation $\lambda =2$. The physical attributes of the water, copper, and titanium dioxide are tabulated in Table 1. The ranges of the pertinent parameters are relevant to the references^37,46,47^.

**Figure 2:**
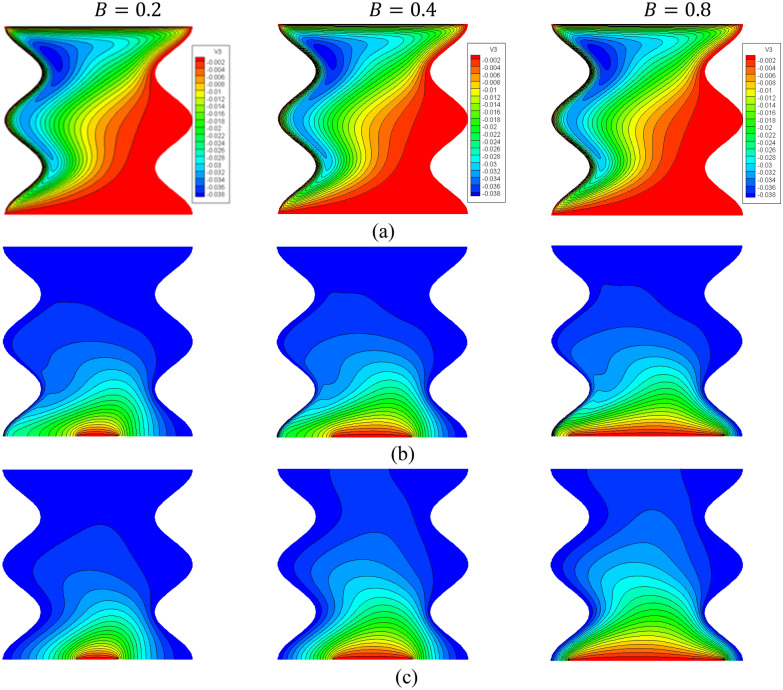
Contours of (**a**) streamlines, (**b**) isotherms of a fluid phase, and (**c**) isotherms of a solid phase below the changes on a partial heat length $B$ for a hybrid nanofluid at ${\phi }_{Cu}={\phi }_{TiO2}=\phi /2,\phi =0.05,Q=1,{R}_{d}=0.5,\varepsilon =0.5,\mathit{Da}=1{0}^{-3},Ha=10,{k}_{fs}=1,D=0.5,{k}_{r}=1,{H}^{*}=10$.

**Figure 3:**
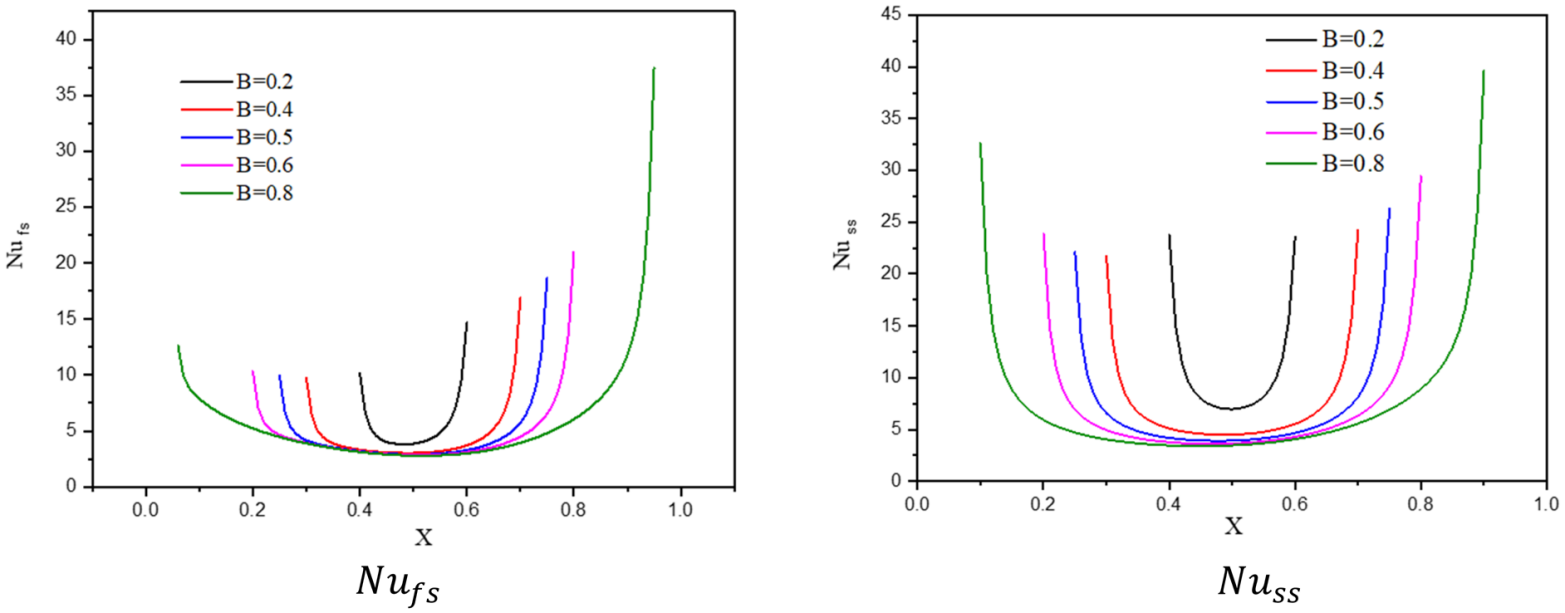
Profiles of $N{u}_{fs}$ and $N{u}_{ss}$, below the changes on a partial heat length $B$ for a hybrid nanofluid at ${\phi }_{Cu}={\phi }_{TiO2}=\phi /2,\phi =0.05,Q=1,{R}_{d}=0.5,\varepsilon =0.5,\mathit{Da}=1{0}^{-3},Ha=10, {k}_{fs}=1,D=0.5,{k}_{r}=1,{H}^{*}=10$.

Figure 2 indicates the contours of the streamlines, isotherms of the fluid/solid phases (two phases) below changes on a partial heat length B for a hybrid nanofluid at ${\phi }_{Cu}={\phi }_{TiO2}=\phi /2,\phi =0.05,Q=1,{R}_{d}=0.5,\varepsilon =0.5,\mathit{Da}=1{0}^{-3},Ha=10,{k}_{fs}=1,D=0.5,{k}_{r}=1,{H}^{*}=10$. In Fig. 2a, there is a little change in the intensity of the streamlines below the changes on a partial heat length *B*. In Fig. 2b,c, the isotherms in the two phases are expanded across a wavy cavity as the partial heat length *B* expanded. Figure 3 shows the sketches of local Nusselt number along with the heater of a fluid phase $N{u}_{fs}$ and of a solid phase $N{u}_{ss}$, below the changes on a partial heat length $B$ for a hybrid nanofluid at ${\phi }_{Cu}={\phi }_{TiO2}=\phi /2,\varphi =0.05,Q=1,{R}_{d}=0.5,\varepsilon =0.5,\mathit{Da}=1{0}^{-3},Ha=10,{k}_{fs}=1,D=0.5,{k}_{r}=1,{H}^{*}=10$. It is noted that the values of $N{u}_{fs}$ and $N{u}_{ss}$ are strongly depending on the distance of a heater. An expansion in a heater length *B* raises the values of $N{u}_{fs}$ and $N{u}_{ss}$. Physically, an increase in the partial heat length B powers the buoyancy force, and consequently the temperature distributions are expanded across a cavity.

**Figure 4:**
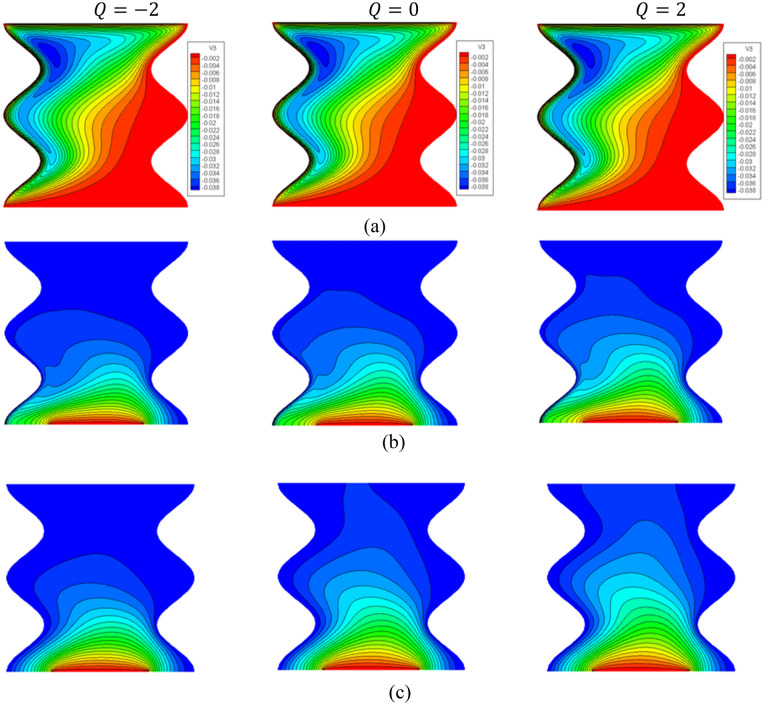
Contours of (**a**) streamlines, (**b**) isotherms of a fluid phase, and (**c**) isotherms of a solid phase below the changes on heat generation/absorption coefficient $Q$ for a hybrid nanofluid at ${\phi }_{Cu}={\phi }_{TiO2}=\phi /2,\phi =0.05,B=0.5,{R}_{d}=0.5,\varepsilon =0.5,\mathit{Da}=1{0}^{-3},Ha=10,{k}_{fs}=1,D=0.5,{k}_{r}=1,{H}^{*}=10$.

**Figure 5:**
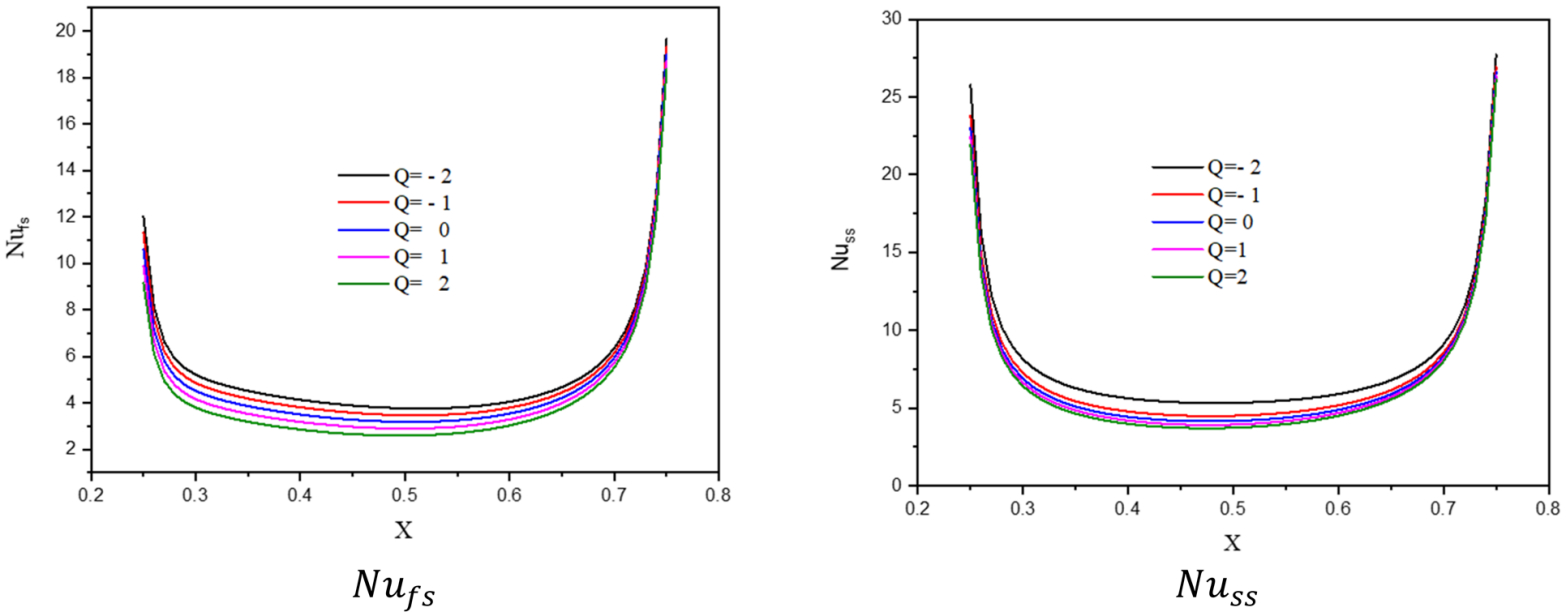
Profiles of the $N{u}_{fs}$ and $N{u}_{ss}$, along the heat source below the changes on heat generation/absorption coefficient $Q$ for a hybrid nanofluid for at ${\phi }_{Cu}={\phi }_{TiO2}=\phi /2,\phi =0.05,B=0.5,{R}_{d}=0.5,\varepsilon =0.5,\mathit{Da}=1{0}^{-3},Ha=10,{k}_{fs}=1,D=0.5,{k}_{r}=1,{H}^{*}=10.$

**Figure 6:**
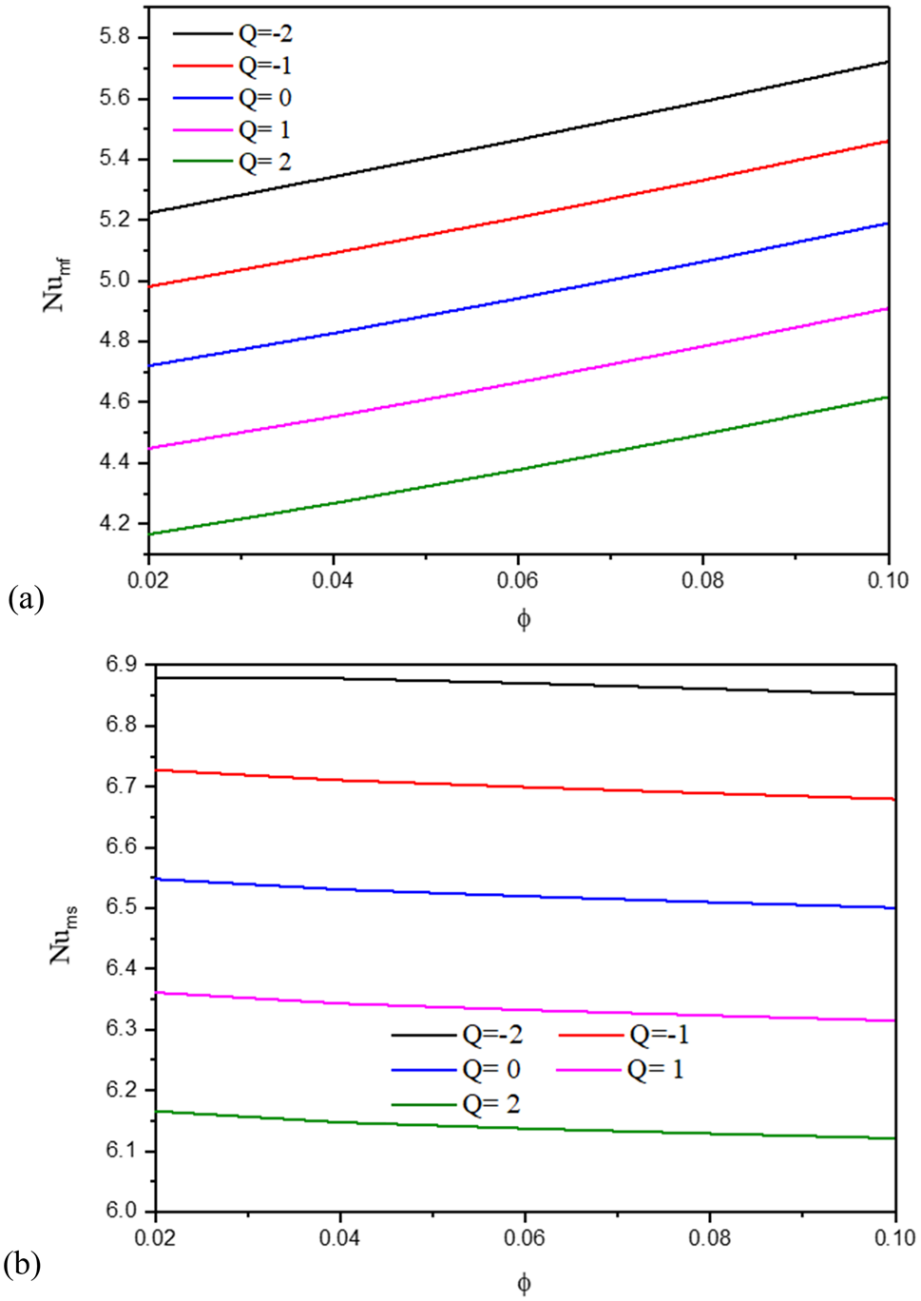
Variation of the $N{u}_{mf}$ (**a**) and $N{u}_{ms}$ (**b**), along a solid volume fraction $\phi$ below the changes on heat generation/absorption coefficient $Q$ for a hybrid nanofluid at ${\phi }_{Cu}={\phi }_{TiO2}=\phi /2,B=0.5,{R}_{d}=0.5,\varepsilon =0.5,\mathit{Da}=1{0}^{-3},Ha=10,{k}_{fs}=1,D=0.5,{k}_{r}=1,{H}^{*}=10.$

Figure 4 introduces the contours of the streamlines, isotherms of the two phases below changes on heat generation/absorption coefficient $Q$ for a hybrid nanofluid at ${\phi }_{Cu}={\phi }_{TiO2}=\phi /2,\phi =0.05,B=0.5,{R}_{d}=0.5,\varepsilon =0.5,\mathit{Da}=1{0}^{-3},Ha=10,{k}_{fs}=1,D=0.5,{k}_{r}=1,{H}^{*}=10$. In Fig. 4a, since the isotherms are formed from the lid velocities in the top and left cavity walls, an increment in the heat generation coefficient $Q$ has little impact on the streamline contours. In Fig. 4b,c, an increase in $Q$ raises the isothermal lines of the two phases within a wavy cavity. The impacts of $Q$ on the $N{u}_{fs}$, and $N{u}_{ss}$, along with a heat source as well as the $N{u}_{mf}$ and $N{u}_{ms}$ are shown in Figs. 5 and 6. The first remark is that an increment in $Q$ declines $N{u}_{fs}$ and $N{u}_{ss}$. Moreover, the values of $N{u}_{mf}$ and $N{u}_{ms}$ are decreasing as $Q$ increases. Growing the concentration of the nanoparticles powers the values of $N{u}_{mf}$ and it has slight influences on $N{u}_{ms}$. The physical reason is returning to the power of a heat generation coefficient $Q$ that enhances the heat transfer in a cavity.

**Figure 7:**
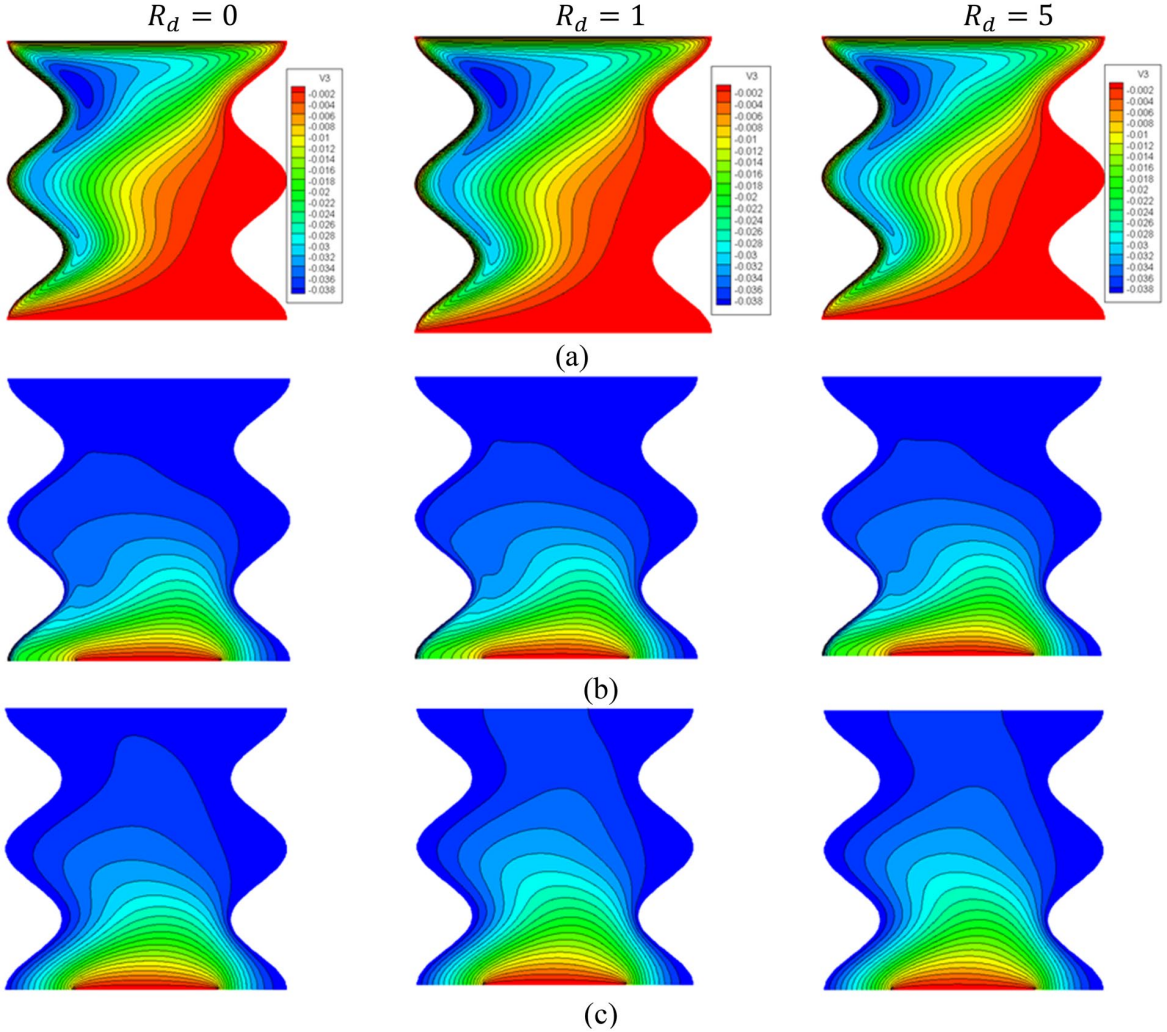
Contours of (**a**) streamlines, (**b**) isotherms of a fluid phase, and (**c**) isotherms of a solid phase below the changes on thermal radiation parameter ${R}_{d}$ for a hybrid nanofluid at ${\phi }_{Cu}={\phi }_{TiO2}=\phi /2,\phi =0.05,B=0.5,Q=1,\varepsilon =0.5,\mathit{Da}=1{0}^{-3},Ha=10,{k}_{fs}=1,D=0.5,{k}_{r}=1,{H}^{*}=10.$

**Figure 8:**
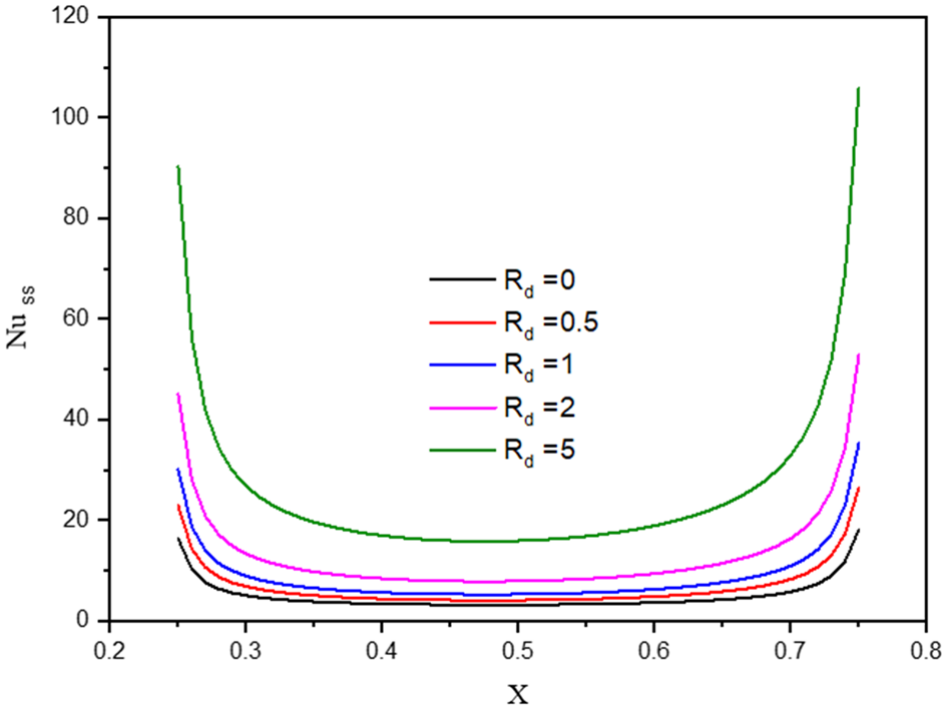
Profiles of $N{u}_{ss}$ along with the heat source of a solid phase below the changes on thermal radiation parameter ${R}_{d}$ for hybrid nanofluid at ${\phi }_{Cu}={\phi }_{TiO2}=\phi /2,\phi =0.05,B=0.5,Q=1,\varepsilon =0.5,\mathit{Da}=1{0}^{-3},Ha=10,{k}_{fs}=1,D=0.5,{k}_{r}=1,{H}^{*}=10.$

**Figure 9:**
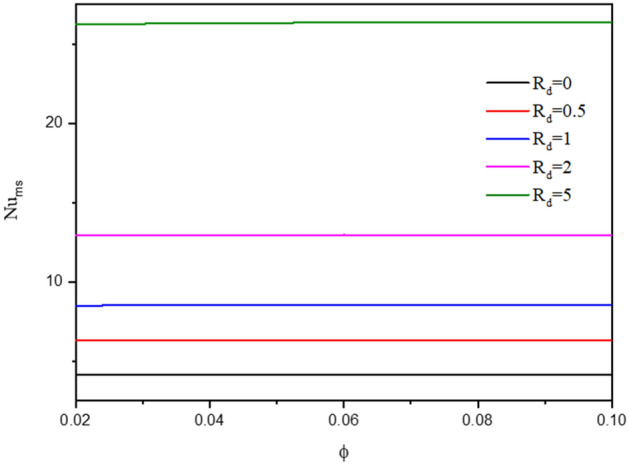
Variation of $N{u}_{ms}$ along $\upphi$ below the changes on the thermal radiation parameter ${R}_{d}$ for a hybrid nanofluid at ${\phi }_{Cu}={\phi }_{TiO2}=\phi /2,B=0.5,Q=1,\varepsilon =0.5,\mathit{Da}=1{0}^{-3},Ha=10,{k}_{fs}=1,D=0.5,{k}_{r}=1,{H}^{*}=10.$

Figure 7 gives the contours of streamlines, isotherms of the two phases below the changes on thermal radiation parameter ${R}_{d}$ for a hybrid nanofluid at ${\phi }_{Cu}={\phi }_{TiO2}=\phi /2,\phi =0.05,B=0.5,Q=1,\varepsilon =0.5,\mathit{Da}=1{0}^{-3},Ha=10,{k}_{fs}=1,D=0.5,{k}_{r}=1, {H}^{*}=10.$ It is remarked that the thermal radiation parameter ${R}_{d}$ has a minor significance on the contours of the streamlines and isothermal lines of a fluid phase within a wavy cavity. Besides, an increment in the thermal radiation parameter ${R}_{d}$ improves the isotherms of a solid phase within a wavy cavity. In general, an increment in the thermal radiation parameter enhances the isotherms. Because ${R}_{d}$ appears in Eq. (11) of a thermal solid phase, the isotherms of a solid phase are enhanced by an increment in ${R}_{d}$. The impacts of a thermal radiation parameter ${R}_{d}$ on the ${Nu}_{ss}$ and ${Nu}_{ms}$ have been shown in Figs. 8 and 9. The values of the ${Nu}_{ss}$ and ${Nu}_{ms}$ are enhancing as ${R}_{d}$ increases.

**Figure 10:**
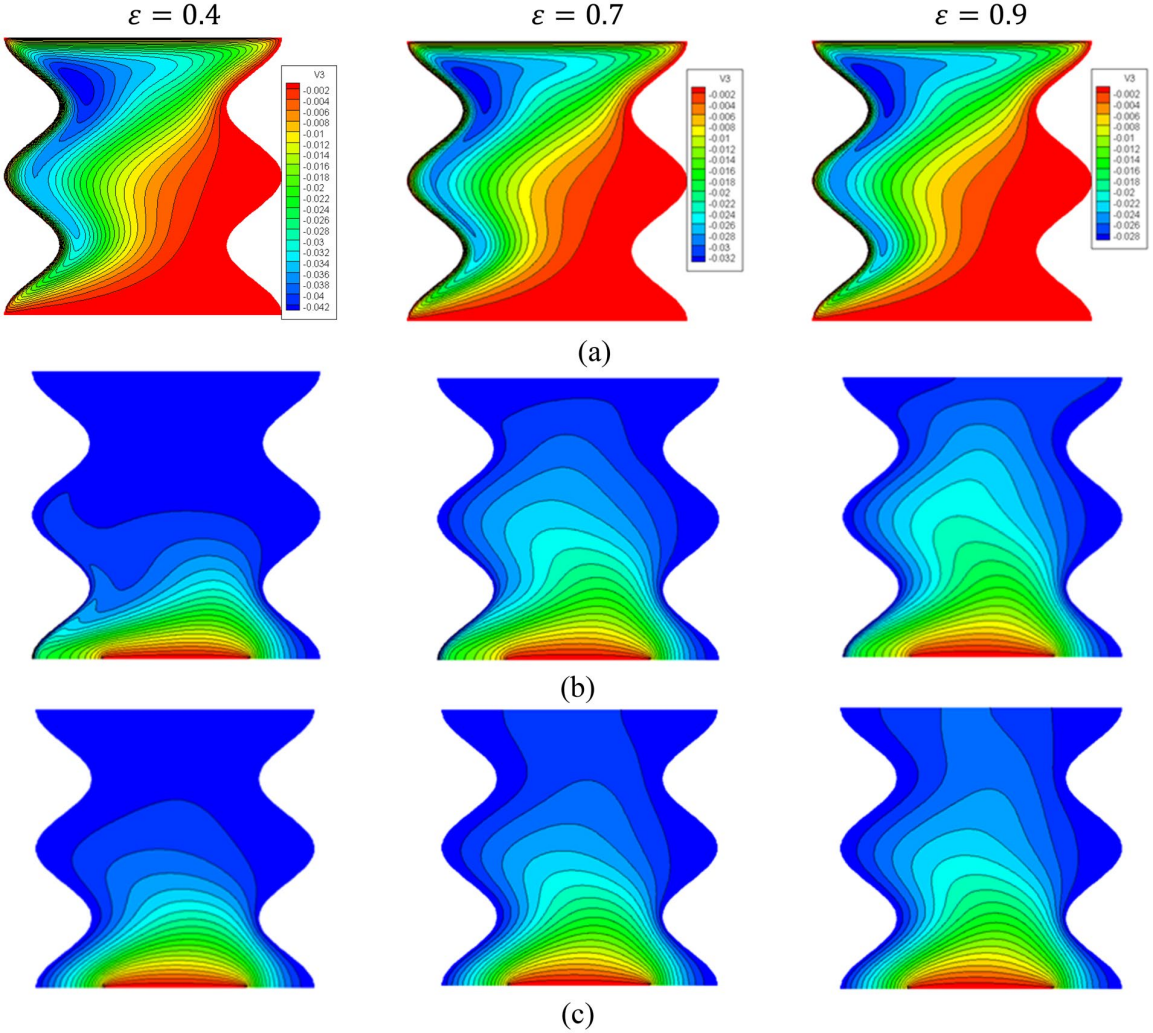
Contours of (**a**) streamlines, (**b**) isotherms of a fluid phase, and (**c**) isotherms of a solid phase below the changes on the porosity parameter $\varepsilon$ for a hybrid nanofluid at ${\phi }_{Cu}={\phi }_{TiO2}=\phi /2,\phi =0.05,\mathit{ B}=0.5,Q=1,{R}_{d}=0.5,\mathit{Da}=1{0}^{-3},Ha=10,{k}_{fs}=1,D=0.5,{k}_{r}=1,{H}^{*}=10.$

Figure 10 introduces the contours of the streamlines, isotherms of the two phases below the changes on the porosity parameter $\varepsilon$ for a hybrid nanofluid at ${\phi }_{Cu}={\phi }_{TiO2}=\phi /2,\varphi =0.05,\mathit{ B}=0.5,Q=1,{R}_{d}=0.5,\mathit{Da}=1{0}^{-3},Ha=10,{k}_{fs}=1,D=0.5,{k}_{r}=1,{H}^{*}=10.$ An increment in a porous parameter $\varepsilon$ from 0.4 to 0.9 declines the absolute of streamlines’ maximum by 33.33%. Moreover, the contours of the isotherms of the two phases are enhanced as a porosity parameter raises.

**Figure 11:**
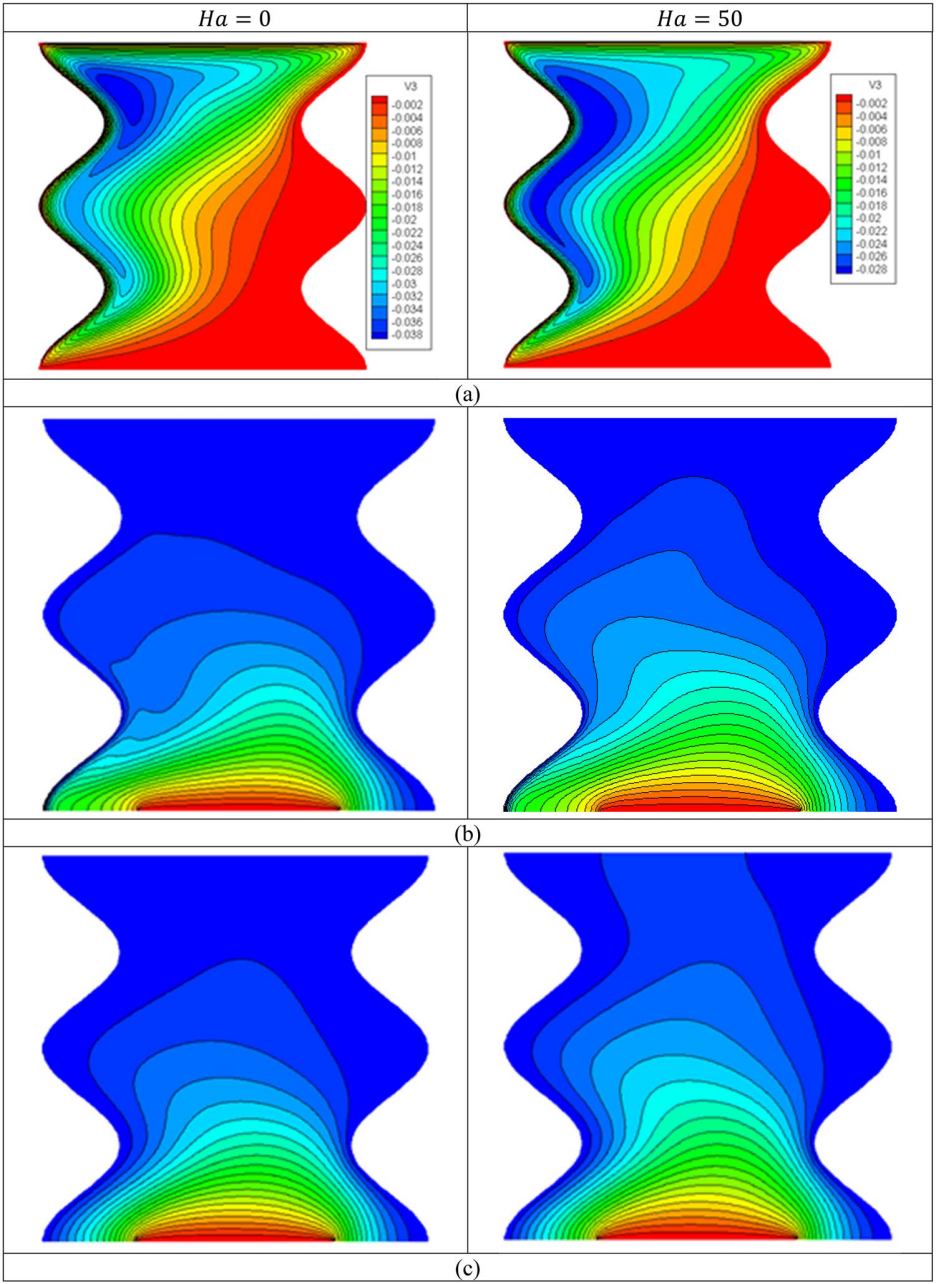
Contours of (**a**) streamlines, (**b**) isotherms of a fluid phase, and (**c**) isotherms of a solid phase below the changes on the Hartmann number $Ha$ for hybrid nanofluid at ${\phi }_{Cu}={\phi }_{TiO2}=\phi /2,\phi =0.05,B=0.5,Q=1,{R}_{d}=0.5,\mathit{Da}=1{0}^{-3},\varepsilon =0.5,{k}_{fs}=1,D=0.5,{k}_{r}=1,{H}^{*}=10$.

**Figure 12:**
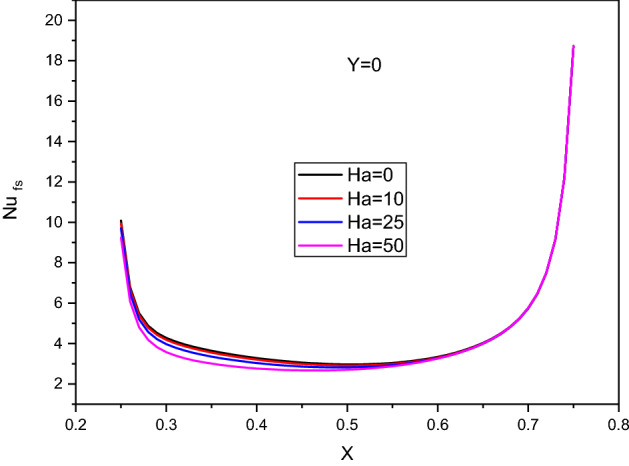
Profiles of $N{u}_{fs}$ along the heat source below the changes on the Hartmann number $Ha$ for hybrid nanofluid at ${\phi }_{Cu}={\phi }_{TiO2}=\phi /2,\phi =0.05, B=0.5, \mathit{ Q}=1, {R}_{d}=0.5,\mathit{Da}=1{0}^{-3},\varepsilon =0.5,{k}_{fs}=1,D=0.5,{k}_{r}=1,{H}^{*}=10.$

**Figure 13:**
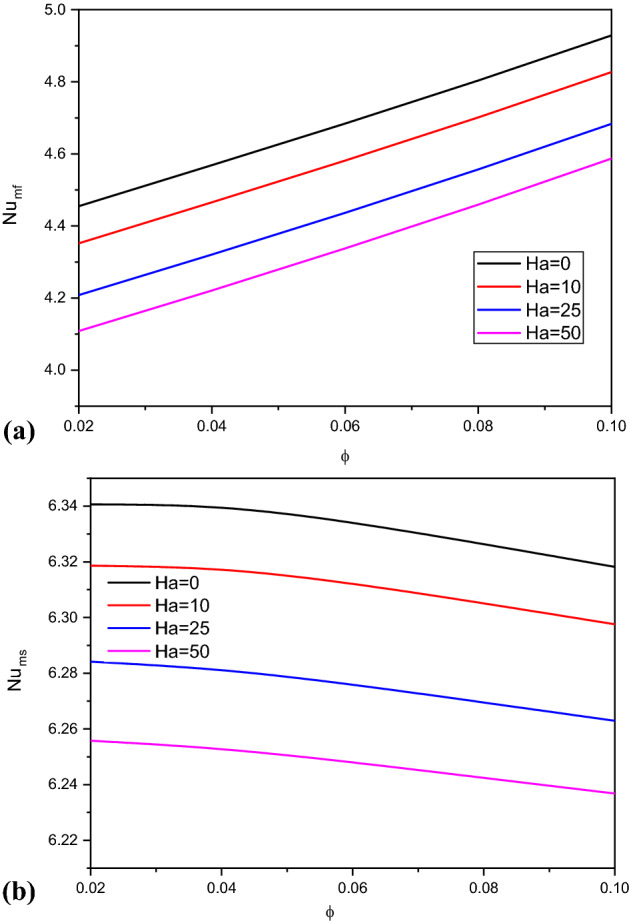
Variation of $N{u}_{mf}$ and $N{u}_{ms}$ along $\upphi$ below the changes on the Hartmann number $Ha$ for a hybrid nanofluid at ${\phi }_{Cu}={\phi }_{TiO2}=\phi /2, B=0.5, Q=1, {R}_{d}=0.5, \mathit{Da}=1{0}^{-3}, \varepsilon =0.5,{k}_{fs}=1,D=0.5,{k}_{r}=1,{H}^{*}=10.$

Figure 11 introduces the contours of the streamlines, isotherms of the two phases below the changes on the Hartmann number $Ha$ for hybrid nanofluid at ${\phi }_{Cu}={\phi }_{TiO2}=\phi /2,\phi =0.05,B=0.5,Q=1,{R}_{d}=0.5,\mathit{Da}=1{0}^{-3},\varepsilon =0.5,{k}_{fs}=1,D=0.5,{k}_{r}=1, { H}^{*}=10$. Physically, an extra $Ha$ generates more Lorentz forces that suppress the flow speed. In Fig. 11a, the absolute of the streamlines’ maximum is decreasing by $26.32\%$ as $Ha$ increases from 0 to 50. In Fig. 11b,c, the isotherms of the two phases are enhanced as the Hartmann number powers. The influences of the Hartmann number on the ${Nu}_{fs}$ along with the heat source, ${Nu}_{mf}$ and ${Nu}_{ms}$ along $\upphi$ have been shown in Figs. 12 and 13. An extension in the Hartmann number reduces ${Nu}_{fs}$, ${Nu}_{mf}$ and ${Nu}_{ms}$. Further, at any value of the Hartmann number, an increase on $\upphi$ enhances ${Nu}_{mf}$.

**Figure 14:**
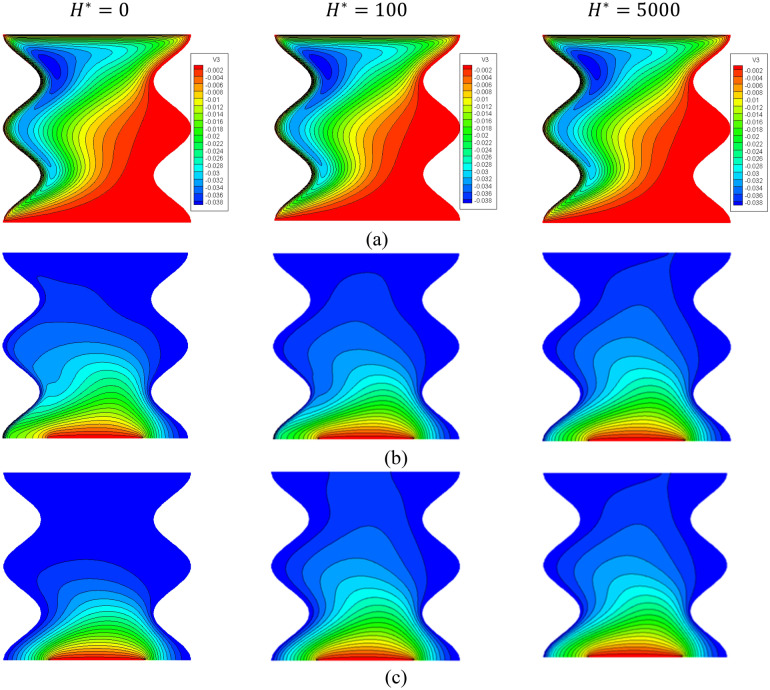
Contours of (**a**) streamlines, (**b**) isotherms of a fluid phase, and (**c**) isotherms of a solid phase below the changes on the inter-phase heat transfer coefficient ${H}^{*}$ for a hybrid nanofluid at ${\phi }_{Cu}={\phi }_{TiO2}=\phi /2,\phi =0.05,B=0.5,Q=1,{R}_{d}=0.5,\mathit{Da}=1{0}^{-3},\varepsilon =0.5,{k}_{fs}=1,D=0.5,{k}_{r}=1,Ha=10.$

**Figure 15:**
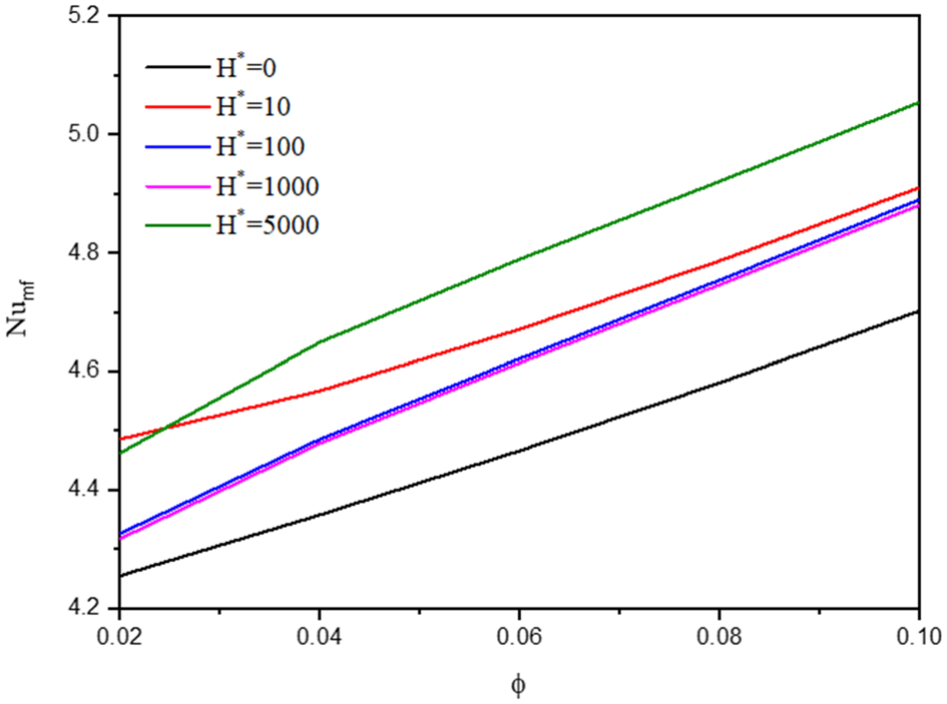
Variation of $N{u}_{mf}$ along $\upphi$ below the changes on the inter-phase heat transfer coefficient ${H}^{*}$ for a hybrid nanofluid at ${\phi }_{Cu}={\phi }_{TiO2}=\phi /2,\mathit{ B}=0.5, Q=1,{R}_{d}=0.5,\mathit{Da}=1{0}^{-3},\varepsilon =0.5,{k}_{fs}=1,D=0.5,{k}_{r}=1,Ha=10.$

Figure 14 shows the contours of the streamlines, isotherms of the two phases below the changes on the inter-phase heat transfer coefficient ${H}^{*}$ for a hybrid nanofluid at ${{k}_{r}=1,\phi }_{Cu}={\phi }_{TiO2}=\phi /2,\phi =0.05,B=0.5,Q=1,{R}_{d}=0.5,\mathit{Da}=1{0}^{-3},\varepsilon =0.5,{ k}_{fs}=1,D=0.5,Ha=10.$ The increment in ${H}^{*}$ has minor influences on the streamlines and improves the isotherms of the two phases. Besides, the average Nusselt number ${Nu}_{mf}$ along $\upphi$ under the changes on ${H}^{*}$ has been shown in Fig. 15. In this figure, there is a fluctuation in ${Nu}_{mf}$ when the value of ${H}^{*}$ between 10 and 1000, while ${H}^{*}=5000$ gives the highest values ${Nu}_{mf}$.

**Figure 16:**
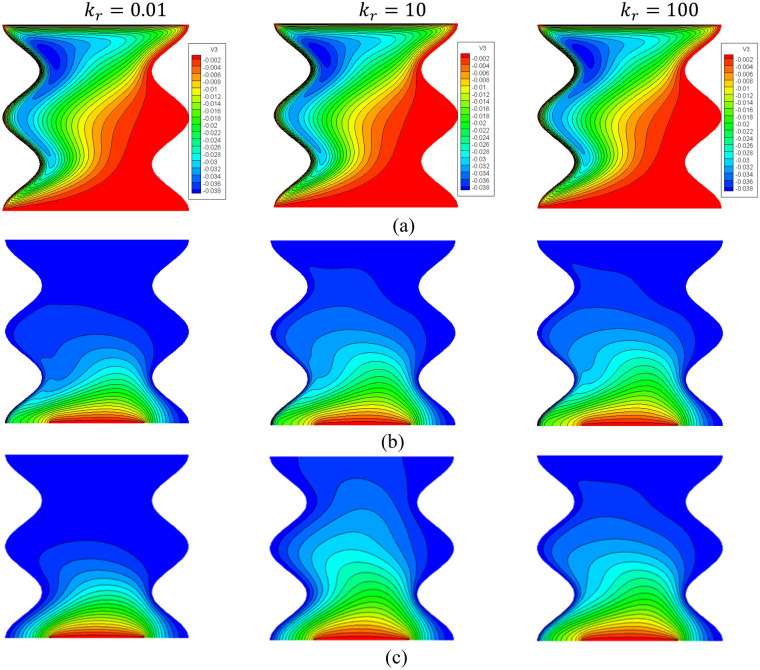
Contours of (**a**) streamlines, (**b**) isotherms of a fluid phase, and (**c**) isotherms of a solid phase below the changes on a modified conductivity ratio ${k}_{r}$ for a hybrid nanofluid at ${\phi }_{Cu}={\phi }_{TiO2}=\phi /2,\phi =0.05,B=0.5,Q=1,{R}_{d}=0.5,\mathit{Da}=1{0}^{-3},\varepsilon =0.5,{k}_{fs}=1,D=0.5,{H}^{*}=10,Ha=10.$

**Figure 17:**
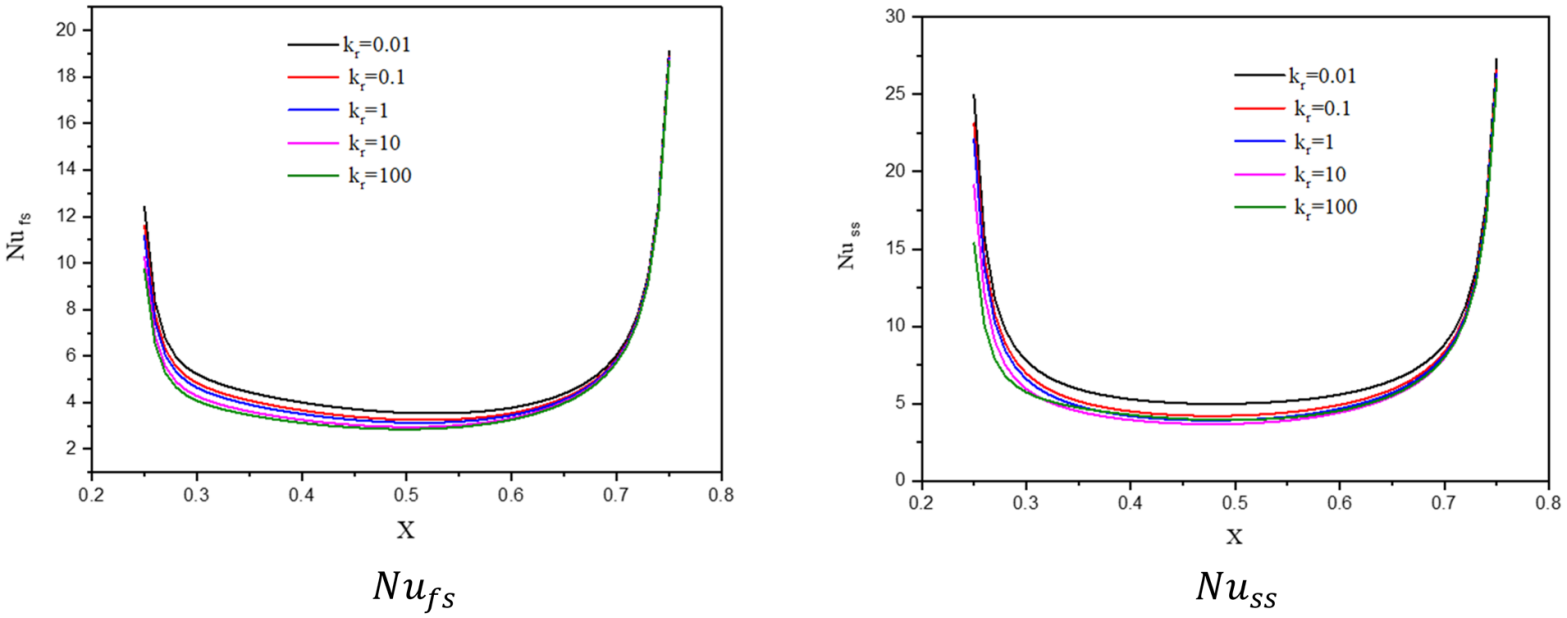
Profiles of $N{u}_{fs}$ and $N{u}_{ss}$ along a heat source below the changes on a modified conductivity ratio ${k}_{r}$ for a hybrid nanofluid at ${\phi }_{Cu}={\phi }_{TiO2}=\phi /2,\phi =0.05, B=0.5, Q=1,{R}_{d}=0.5,\mathit{Da}=1{0}^{-3},\varepsilon =0.5,{k}_{fs}=1,D=0.5,{H}^{*}=10,Ha=10.$

**Figure 18:**
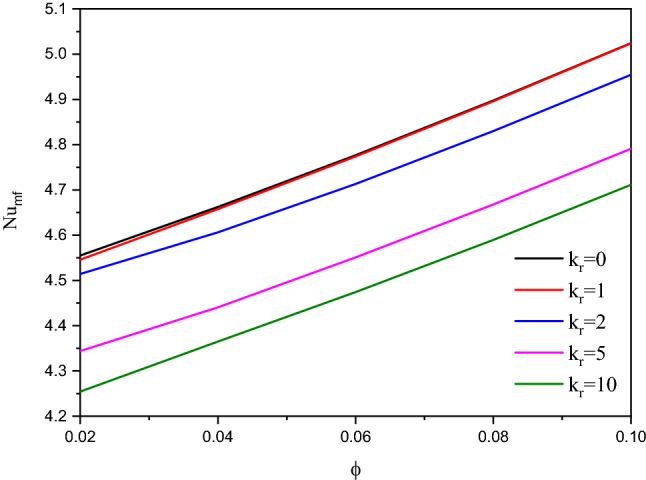
Variation of $N{u}_{mf}$ below the changes on a modified conductivity ratio ${k}_{r}$ for a hybrid nanofluid at ${\phi }_{Cu}={\phi }_{TiO2}=\phi /2, B=0.5, Q=1, {R}_{d}=0.5, \mathit{Da}=1{0}^{-3},\varepsilon =0.5,{k}_{fs}=1, D=0.5, {H}^{*}=10,Ha=10$.

The effects of a modified conductivity ratio ${k}_{r}$ on the streamlines, isotherms of the two phases, local and average Nusselt number at ${\phi }_{Cu}={\phi }_{TiO2}=\phi /2,\phi =0.05, B=0.5, Q=1, {R}_{d}=0.5,\mathit{Da}=1{0}^{-3},\varepsilon =0.5,{k}_{fs}=1,D=0.5,{H}^{*}=10,Ha=10$ have been shown in Figs. 16, 17 and 18. The first remark is that the variations on the modified conductivity ratio ${k}_{r}$ have minor effects on streamlines and isotherms of a fluid phase contours, whilst the isotherms of a solid phase are significantly affected. In Figs. 17 and 18, a raise in ${k}_{r}$ declines the values of ${Nu}_{fs}$, ${Nu}_{ss}$ and ${Nu}_{mf}$. Physically, an increase in the thermal conductivity ratio ${k}_{r}$ declines the thermal challenge of porous media.

**Figure 19:**
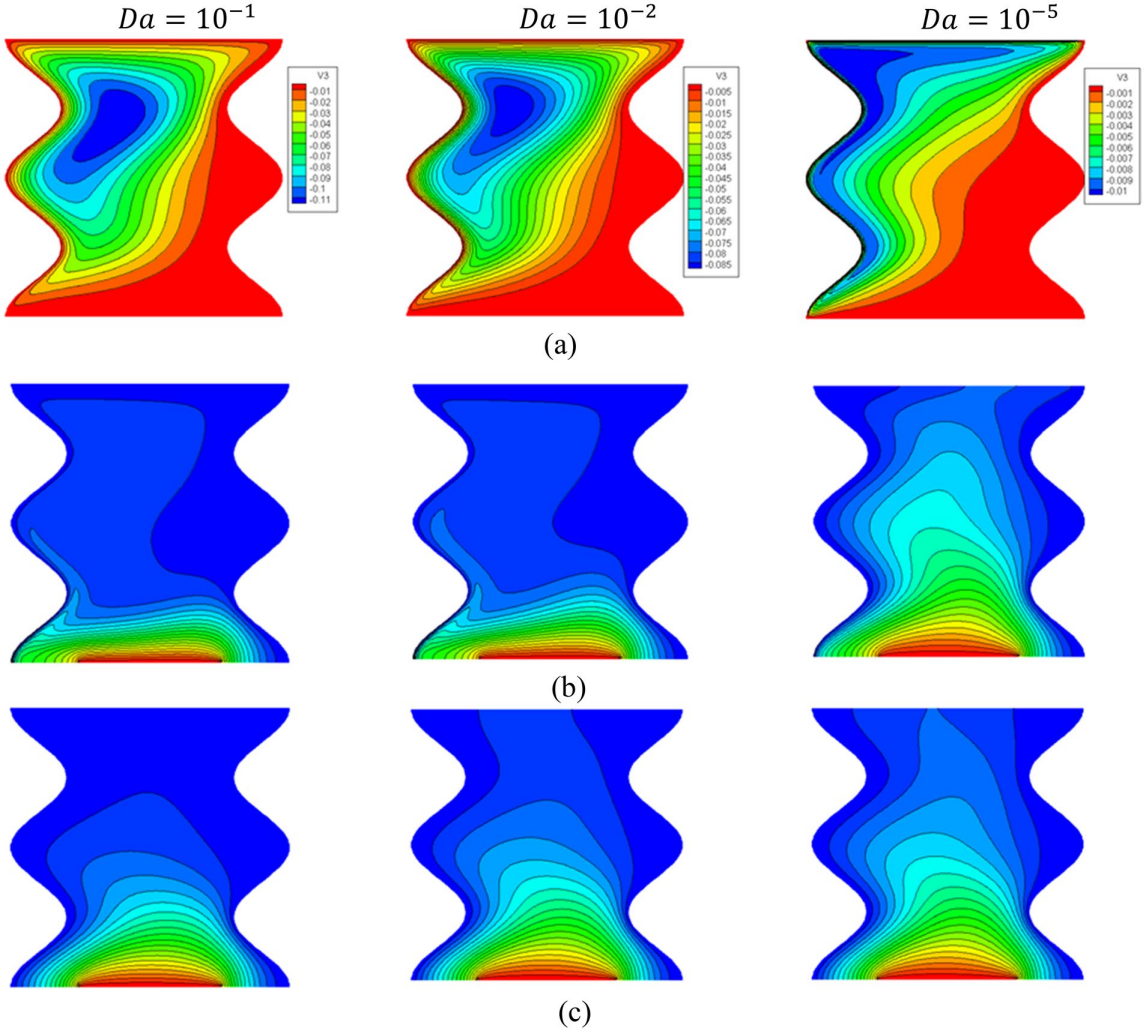
Contours of (**a**) streamlines, (**b**) isotherms of a fluid phase, and (**c**) isotherms of a solid phase below the changes on Darcy parameter for a hybrid nanofluid at ${\phi }_{Cu}={\phi }_{TiO2}=\phi /2, \phi =0.05, B=0.5,Q=1,{R}_{d}=0.5,{k}_{r}=1,\varepsilon =0.5,{k}_{fs}=1,D=0.5, {H}^{*}=10,Ha=10.$

**Figure 20:**
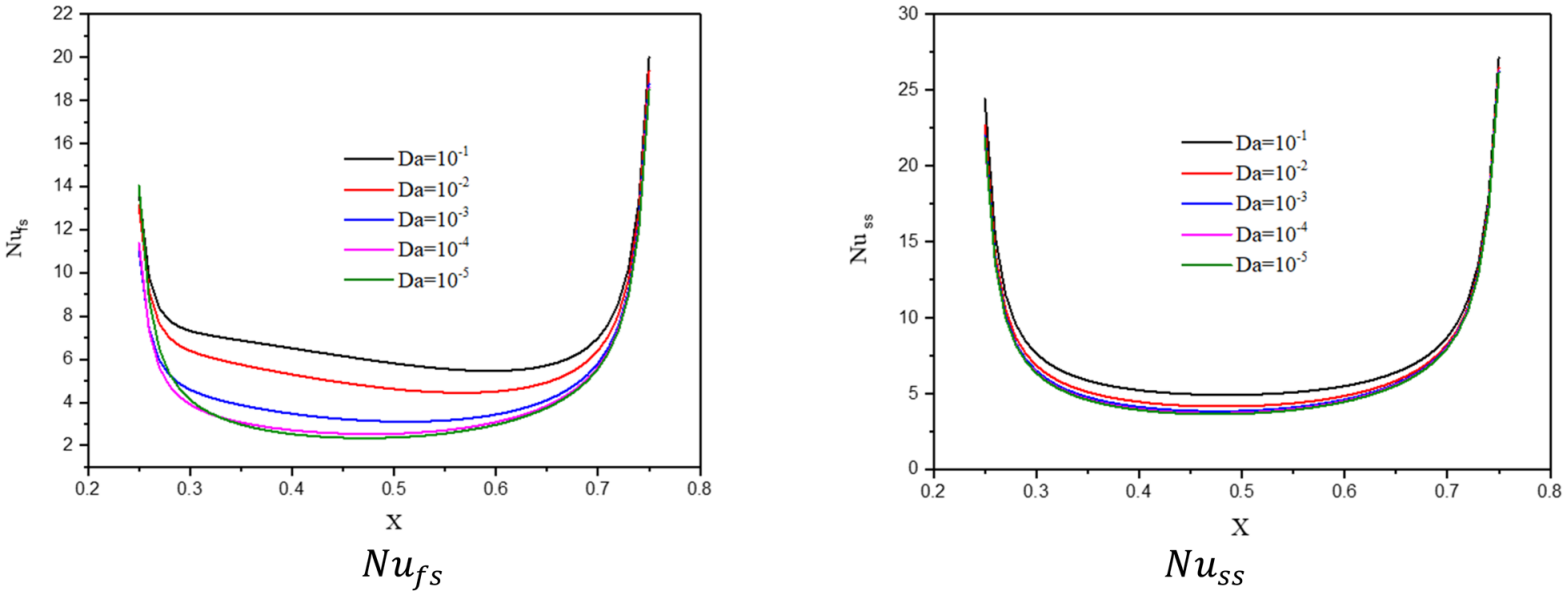
Profiles of $N{u}_{fs}$ and $N{u}_{ss}$ below the changes on Darcy parameter for a hybrid nanofluid at ${\phi }_{Cu}={\phi }_{TiO2}=\phi /2,\phi =0.05,B=0.5,Q=1,{R}_{d}=0.5,{k}_{r}=1,\varepsilon =0.5,{k}_{fs}=1,D=0.5,{H}^{*}=10,Ha=10.$

**Figure 21:**
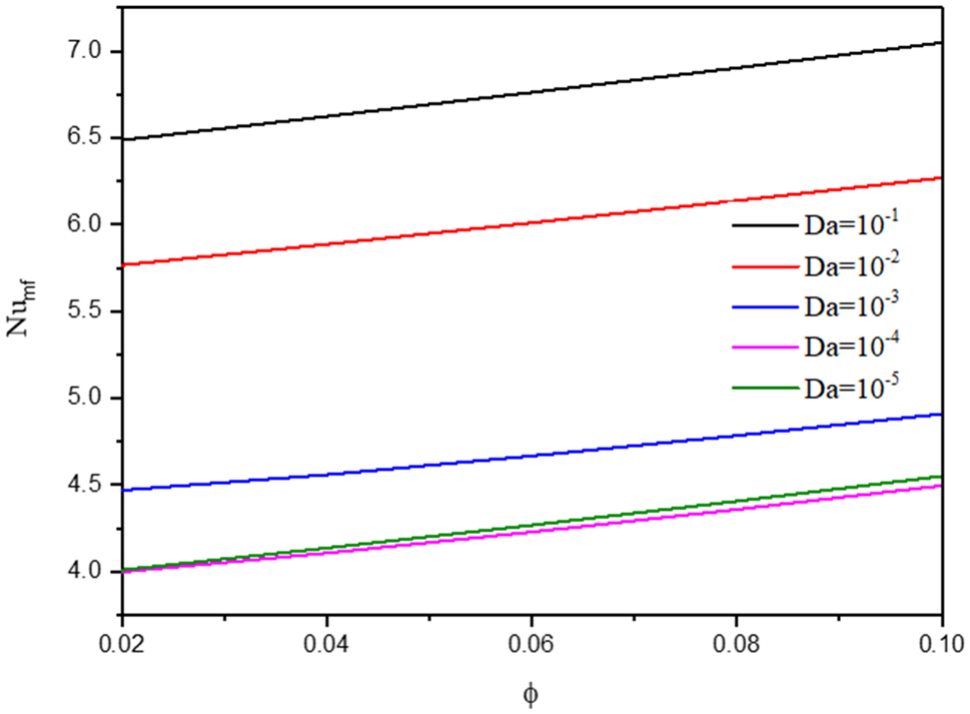
Variation of $N{u}_{mf}$ below the changes on Darcy parameter for a hybrid nanofluid at ${\phi }_{Cu}={\phi }_{TiO2}=\phi /2,B=0.5,Q=1,{R}_{d}=0.5,\mathit{Da}=1{0}^{-3},\varepsilon =0.5, {k}_{fs}=1, D=0.5, {H}^{*}=10, Ha=10.$

The influences of a Darcy parameter on the streamlines, isotherms of the two phases, local and average Nusselt number have been shown in Figs. 19, 20 and 21. A reduction in a Darcy parameter provides more resistance of the porous media for the nanofluid flow. As a result, as the Darcy parameter lowers to 10–5 from 10 to 1, the absolute standards of a maximum of streamlines are lessening by 90.91%. Thus, a decrease of the Darcy parameter is enhancing the isotherms of the two phases within a wavy cavity. In Figs. 20 and 21, a decline in the Darcy parameter lowers the values of local and average Nusselt number.

**Figure 22:**
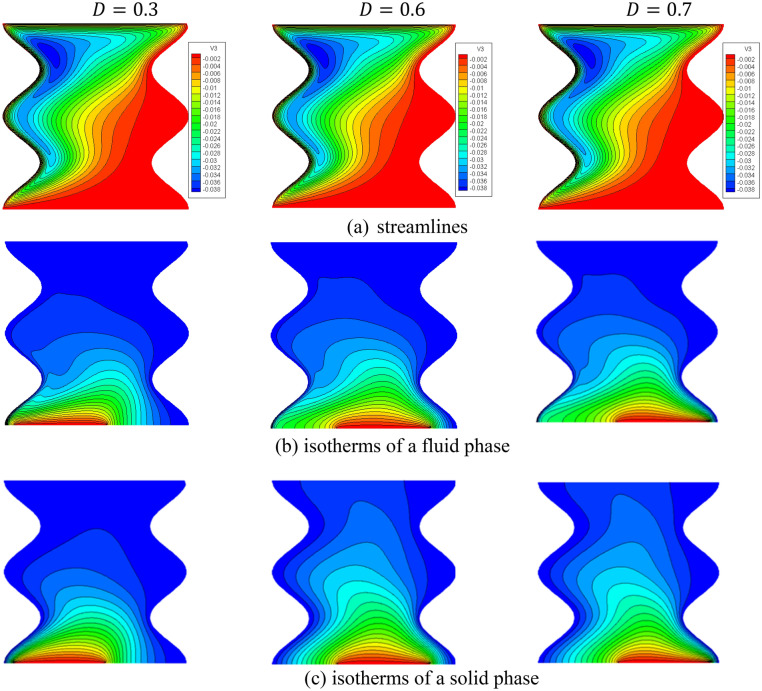
Contours of (**a**) streamlines, (**b**) isotherms of a fluid phase, and (**c**) isotherms of a solid phase below the changes on a heat source position $D$ for a hybrid nanofluid at ${\phi }_{Cu}={\phi }_{TiO2}=\phi /2, \phi =0.05, B=0.5,Q=1,{R}_{d}=0.5,{k}_{r}=1,\varepsilon =0.5,{k}_{fs}=1,Da=1{0}^{-3}, {H}^{*}=10, Ha=10.$

**Figure 23:**
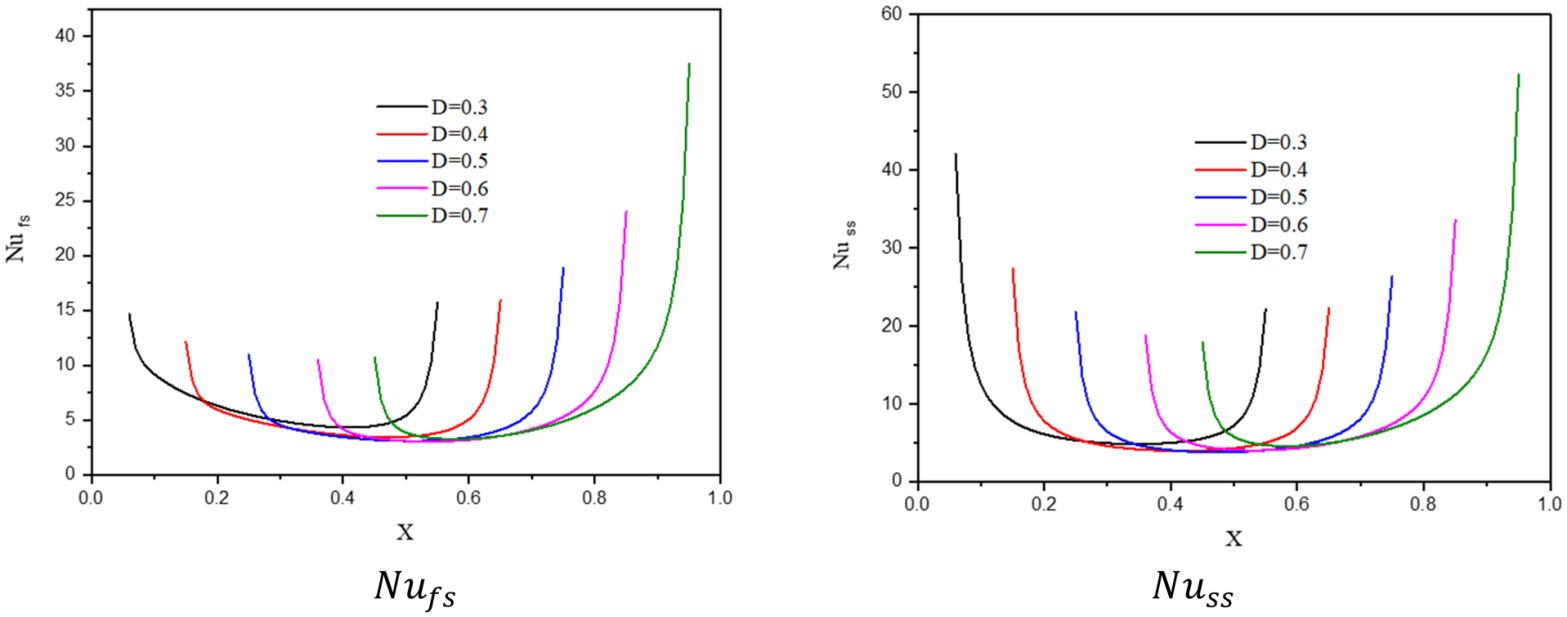
Profiles of $N{u}_{fs}$ and $N{u}_{ss}$ along the heat source below the changes on a heat source position $D$ for a hybrid nanofluid at ${\phi }_{Cu}={\phi }_{TiO2}=\phi /2,\phi =0.05, B=0.5, Q=1, {R}_{d}=0.5,{k}_{r}=1,\varepsilon =0.5,{k}_{fs}=1,Da=1{0}^{-3},{\mathit{ H}}^{*}=10,Ha=10.$

**Figure 24:**
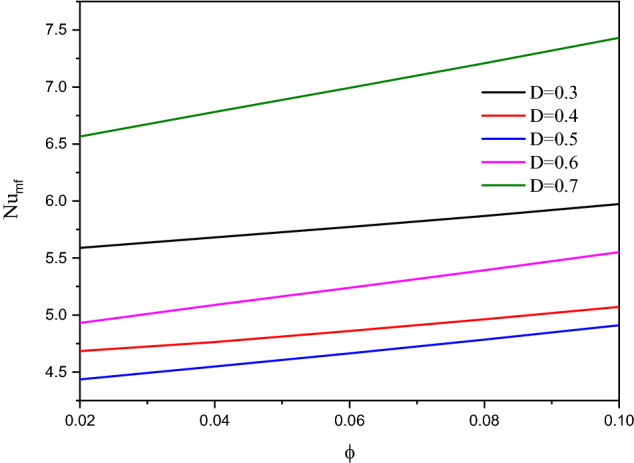
Variation of $N{u}_{mf}$ below the changes on a heat source position $D$ for a hybrid nanofluid at ${\phi }_{Cu}={\phi }_{TiO2}=\phi /2,B=0.5,Q=1,{R}_{d}=0.5,{k}_{r}=1,\varepsilon =0.5, {k}_{fs}=1, Da=1{0}^{-3},{H}^{*}=10,Ha=10$.

Figure 22 shows the contours of streamlines, isotherms of the two phases below the changes on a heat source position $D$ for a hybrid nanofluid at ${\phi }_{Cu}={\phi }_{TiO2}=\phi /2,\phi =0.05,B=0.5,Q=1,{R}_{d}=0.5,{k}_{r}=1,\varepsilon =0.5,{k}_{fs}=1,Da=1{0}^{-3},{H}^{*}=10,Ha=10.$ It is clear that when the position of a heat source is changing from the left side $(D=0.3)$ to the right side $(D=0.7)$ of a wavy cavity, little variations are occulting in the streamlines contours and the isotherms of the two phases are significantly influenced. The impacts of a heat source position $D$ on the values of ${Nu}_{fs}$, ${Nu}_{ss}$ and ${Nu}_{mf}$ have been introduced in Figs. 23 and 24. Here, changing the location of a heat source towards the right side of an undulating cavity raises the values of ${Nu}_{fs}$, ${Nu}_{ss}$ and ${Nu}_{mf}$. Thus, the values of ${Nu}_{ss}$ and ${Nu}_{mf}$ are higher when the heater is located nearly to a left side $(D=0.3)$ and a right side $(D=0.7)$ of an undulating cavity.

## Conclusion

This study is introducing the first attempt in solving the mixed convection of hybrid nanofluids within an undulating porous cavity under the LTNE condition. The contours of the streamlines, isotherms of fluid/solid phases as well as the profiles of local and average Nusselt number on the fluid/solid phases under the variations of the key parameters like partial heat length (B)—position (D), modified conductivity ratio ${k}_{r}$, coefficient of heat generation/absorption $Q$, thermal radiation parameter ${R}_{d}$, Hartmann number $Ha$, porosity parameter $\varepsilon$, an inter-phase heat transfer coefficient ${H}^{*}$, Darcy parameter $Da$, and hybrid nanofluid parameter $\upphi$ have been obtained. The remarkable points could be concluded as:

- The length and position of the partial heat are acting effectively in adjusting the features of heat transfer and nanofluid movements inside an undulating cavity.
- The isotherms strength of a solid phase is mounting as the heat generation/absorption coefficient and thermal radiation parameter are increased.
- Augmentation in the Hartmann number from 0 to 50 lessens the streamlines’ maximum by $26.32\%$ and reduces ${Nu}_{fs}$, ${Nu}_{mf}$ and ${Nu}_{ms}$. Physically, increasing $Ha$ augments the magnetic Lorentz force which reduces the nanofluid movements.
- The isotherms of a solid phase are significantly affected by the variations on ${H}^{*}$ and ${k}_{r}$.
- The values of ${Nu}_{mf}$ are enhancing according to an increase in the nanoparticles concentration by increasing $\upphi$.
- The intensity of the isotherms of the two phases is enhancing as a porous parameter $\varepsilon$ expands.
- High resistance of a porous medium (smaller values of a Darcy parameter) provides a reduction in the nanofluid movements and enhances the isotherms of the two phases within an undulating cavity.

## Abbreviations

$$B$$: Heat length, *m*
${B}_{0}$: Magnetic field strength
$$g$$: Gravitational acceleration, m/s^2^
$$Da$$: Darcy number
$D$: Position of a heat source
${H}^{*}$: Inter-phase heat transfer coefficient
$$Ha$$: Hartmann number
$$Gr$$: Grashof number
${k}_{r}$: Modified conductivity ratio
$$Nu$$: Nusselt number
$$P$$: Pressure, N/m^2^
$$Pr$$: Prandtl number
$$Re$$: Reynolds number
${R}_{d}$: Thermal radiation parameter
$$Ri$$: Richardson parameter
${Q}_{0}$: Heat generation/absorption coefficient
$$T$$: Temperature, K
$$u, v$$: Velocity components, m/s
$U, V$: Dimensionless velocity components
$$x, y$$: Cartesian coordinates, m
$$X, Y$$: Dimensionless coordinates

## Greek symbols

$$\alpha$$: Inclination angle
$\beta$: Thermal expansion coefficient, K^−1^
$$\varepsilon$$: Porosity
$\upphi$: Nanoparticle volume fraction
$\Phi$: Magnetic field inclination angle
$$\mu$$: Dynamic viscosity
$$\theta$$: Dimensionless temperature
$$\nu$$: Kinematic viscosity, m^2^/s
$$\rho$$: Density, kg/m^3^

## Subscripts

$$f$$: Base fluid
$$s$$: Porous
$$hn$$: Hybrid nanofluid
$$c$$: Cold
$$h$$: Hot
